# Novel Quaternary Ammonium Urethane-Dimethacrylates for Copolymers with Low Water Sorption and Solubility

**DOI:** 10.3390/molecules30040769

**Published:** 2025-02-07

**Authors:** Patryk Drejka, Patrycja Kula, Izabela Barszczewska-Rybarek

**Affiliations:** Department of Physical Chemistry and Technology of Polymers, Faculty of Chemistry, Silesian University of Technology, Strzody 9 Str., 44-100 Gliwice, Poland; patryk.drejka@polsl.pl (P.D.); patrycja.kula@polsl.pl (P.K.)

**Keywords:** urethane-dimethacrylates, quaternary ammonium groups, dental materials, composite matrix, water sorption, water solubility

## Abstract

Six novel urethane-dimethacrylates with quaternary ammonium groups (QAUDMAs) were successfully synthesized from 2-(methacryloyloxy)ethyl-2-hydroxyethylmethylalkylammonium bromide (QAHAMA-n, where n was 8 and 10) and diisocyanate (isophorone diisocyanate (IPDI), 4,4′-methylenedicyclohexyl diisocyanate (CHMDI), and 4,4′-diphenylmethane diisocyanate (MDI)). Their chemical structures were confirmed through nuclear magnetic resonance spectroscopy (NMR) and Fourier transform infrared spectroscopy (FTIR). The refractive index (RI) and density (d_m_) were also determined. The novel QAUDMAs were compounded with common dental dimethacrylates and subsequently photopolymerized. The resulting copolymers, comprising QAUDMA 40 wt.%, bisphenol A glycerolate dimethacrylate (Bis-GMA) 40 wt.%, and triethylene glycol dimethacrylate (TEGDMA) 20 wt.%, were tested for water sorption (WS) and solubility (SL). The WS and SL values decreased following these orderings based on the diisocyanate: IPDI > CHMDI > MDI for WS, and MDI > CHMDI > IPDI for SL. The WS values ranged from 11.50 to 13.82 µg/mm^3^, and were significantly lower than the recommended maximum for dental materials, 40 µg/mm^3^. The SL values that met the recommended maximum, 7.5 µg/mm^3^, ranged from 2.67 to 6.75 µg/mm^3^. Only the copolymer having the QAHAMA-8- and MDI-derived QAUDMA had the SL slightly exceeding 7.5 µg/mm^3^, at 7.89 µg/mm^3^.

## 1. Introduction

Tooth decay is the most common oral disease worldwide [1]. The formation of biofilm, which consists of agglomerates of pathogenic bacteria (primarily *Streptococcus mutans*) and fungi (primarily *Candida albicans*), is the leading cause of this disease [2,3,4,5]. The common strategies to control human biofilm include: (i) introduction of appropriate eating habits with limited sugar intake; (ii) improvement of oral hygiene; (iii) mechanical biofilm removal by brushing, interdental cleaning, and scaling; (iv) chemical biofilm control by using a fluoride toothpaste and dental rinses with antibacterial ingredients [6,7]. However, the abovementioned solutions only have a temporary antibacterial effect [8]. Using dental composite restorative materials (DCRMs) with antibacterial properties would delay biofilm formation or reduce its scale [9,10,11,12,13,14,15,16,17,18,19,20,21,22,23]. To address this problem, scientists usually modify existing DCRMs by introducing antibiotics (such as ciprofloxacin) [9,10], antiseptics (such as chlorohexidine salts [16,20]), polyethyleneimine-based nanoparticles having quaternary ammonium (QA) groups [14], and nanoparticles of inorganic compounds, such as silver or titanium dioxide [11,13,20,23]. The primary aim of modifying dental materials is to delay or, ideally, prevent the development of secondary caries. However, the antibacterial effect should be limited to the surfaces of treated teeth, which are susceptible to the accumulation of pathogenic bacteria, particularly in marginal gaps. This approach is beneficial for maintaining a natural oral microbiome. Physical modification of dental materials has two significant drawbacks. First, the effectiveness of the antibacterial agents diminishes over time due to dental abrasion and intentional leaching of a biocide. Second, when these compounds enter the saliva, they may exert an antibacterial effect throughout the entire oral cavity and potentially in other distant organs, disrupting the natural microflora both in the mouth and throughout the body [24,25]. Given these concerns, modifying DCRMs’ matrices by copolymerizing common dental dimethacrylate monomers with those containing bioactive quaternary ammonium (QA) groups appears to be a more promising approach. Such materials could potentially maintain their antibacterial properties over time while minimizing adverse effects [26,27,28,29,30,31,32]. This approach is widely investigated and includes: (i) designing structures and synthesis routes of novel dimethacrylate monomers with QA functionalities (QADMAs); (ii) copolymerizing QADMAs with common dental dimethacrylates (DMAs), such as bisphenol A glycerolate dimethacrylate (Bis-GMA), urethane-dimethacrylate monomer (UDMA), and triethylene glycol dimethacrylate (TEGDMA); (iii) testing the physicochemical, mechanical, and antimicrobial properties of the resulting copolymers [26,27,28,29,30,31,32].

**Figure 1:**
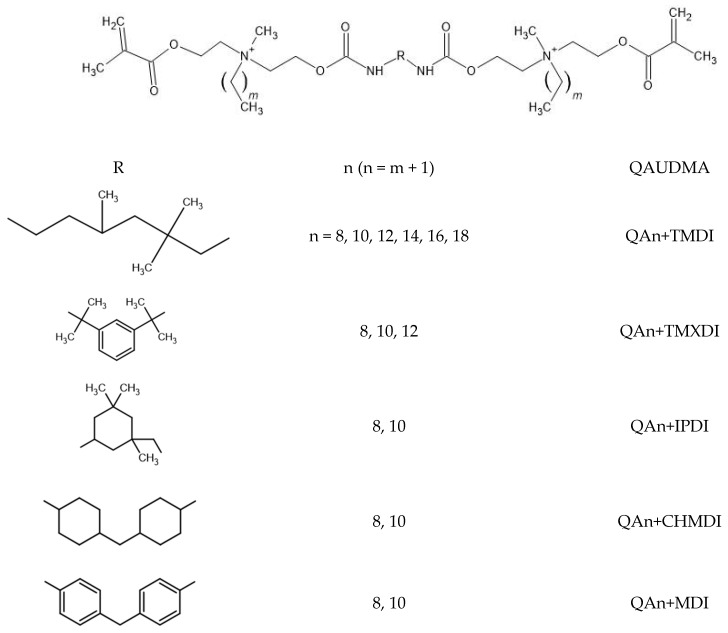
The chemical structure of novel QAUDMA monomers (QAn+IPDI, QAn+CHMDI, QAn+MDI) as well as those described in the literature (QAn+TMDI [42], QAn+TMXDI [44]), having the diisocyanate-derived core, and two quaternary ammonium groups located in the methacrylate terminated wings.

The research explored QADMAs derived from N-methyldiethanolamine (MDEA), N,N-dimethylaminoethyl methacrylate (DMAEMA), and diethylamine (DEA) [33,34,35,36,37,38,39,40,41,42,43,44,45]. The copolymers of QADMAs with dental DMAs usually demonstrated significant antibacterial activity. Huang et al. introduced 10 wt.% of the DMAEMA-derived monomer, having one central QA group with hexadecyl substituent in the Bis-GMA/TEGDMA composition, and observed a significantly decreased number of *S. mutans* colonies on the copolymer surface [34]. Liang et al. added 10 and 20 wt.% of the MDEA-based quaternary ammonium urethane-dimethacrylate, with two QA groups having hexadecyl substituent (QA16+MDEA) into the Bis-GMA/TEGDMA composition, which resulted in a copolymer with high antibacterial activity against *S. mutans* [35]. Interestingly, Huang et al. also introduced from 5 to 20 wt.% of QA16+MDEA to the UDMA/tricyclodecane dimethanol diacrylate system and only observed moderate antibacterial activity of the resulting copolymer, which was the highest for QA16+MDEA 17 wt.% [36]. Liang et al. achieved antibacterial copolymers composed of 50 wt.% of TEGDMA and 50 wt.% of the MDEA-derived monomers, having one central QA group substituted with the N-alkyl chain and having from twelve to sixteen carbon atoms and isophorone diisocyanate moieties located in the wings (QAn+MDEA+IPDI, where n = 12–16) [37]. Huang et al., in another study, developed a copolymer with antibacterial properties by introducing 30 wt.% of QA12+MDEA+IPDI in Bis-GMA/TEGDMA composition [38]. Manouchehri et al. demonstrated that the addition of 1 wt.% of another DMAEMA-derived QADMA, having two QA groups linked with tetramethylene or hexamethylene chain, in the Tetric N-Bond dental adhesive was sufficient to produce a material with antibacterial activity against *S. mutans* [39]. Makvandi et al. modified the Bis-GMA/TEGDMA copolymer by introducing 15 wt.% of the Bis-GMA quaternary ammonium analog having two DEA-derived QA groups with hexyl substituents (QA6+DEA+Bis-GMA). The resulting copolymer demonstrated antibacterial activity against *Escherichia coli*, *Pseudomonas aeruginosa*, and *Staphylococcus aureus* [40]. Zhang et al. showed significant antibacterial activity against *S. mutans* for the Bis-GMA/TEGDMA copolymer containing 10 wt.% of the DMAEMA-derived QADMA with a betulin core [41]. In 2019, our research group proposed another QADMA monomer approach, the MDEA- and 2,2,4-trimethylhexamethylene diisocyanate (TMDI)-derived analogs of the UDMA monomer. They had QA groups substituted with the N-alkyl chains of eight to eighteen carbon atoms (QAn+TMDI, where n = 8, 10, 12, 14, 16, and 18, Figure 1) [42]. The copolymerization of 40 wt.% of QA8+TMDI, QA10+TMDI, and QA12+TMDI with Bis-GMA/TEGDMA composition resulted in polymers with antibacterial activity against *S. aureus* and *E. coli* [43]. We found that the shorter the N-alkyl substituent, the higher the antibacterial activity. We also synthesized another three quaternary ammonium urethane-dimethacrylates (QAUDMAs) utilizing 1,3-bis(1-isocyanato-1-methylethyl)benzene (QAn+TMXDI, where n = 8, 10, and 12, Figure 1) [44,45]. The resulting copolymers of QAn+TMXDI 20 wt.% with Bis-GMA 40 wt.%, UDMA 20 wt.%, and TEGDMA 20 wt.% showed slight antibacterial activity against *S. aureus* and *E. coli* [44,45], which increased as UDMA was completely replaced with QAn+TMXDI, i.e., the latter was used in 40 wt.% [45].

The examples above demonstrate that using QADMAs as components of DMA copolymers has a high potential for developing a new class of DCRM matrices with antibacterial activity. However, the physicochemical and mechanical properties of these polymers often deteriorate. In particular, water sorption (WS) and solubility (SL) significantly increased.

Liang et al. observed that the WS and SL values of the QAn+MDEA+IPDI/TEGDMA 50 wt.%/50 wt.% copolymers were 1.9 and 3.9 times higher than those of the Bis-GMA/TEGDMA 50 wt.%/50 wt.% reference copolymer (WS = 4.10%, and SL = 2.31%) [37]. Huang et al. decreased the QA12+MDEA+IPDI content in the copolymer by its partial replacement with Bis-GMA, which caused less significant decreases in the WS and SL, being, respectively, 1.5 and 2.0 times higher in comparison to the Bis-GMA/TEGDMA 50 wt.%/50 wt.% reference copolymer [38]. Makvandi et al. demonstrated that the introduction of QA6+DEA+Bis-GMA 15 wt.% in the Bis-GMA/TEGDMA 50 wt.%/50 wt.% mixture resulted in 2.0 times higher WS and 1.2 times higher SL compared to the Bis-GMA/TEGDMA 50 wt.%/50 wt.% reference (WS = 1.55%, SL = 0.47%) [40]. The Bis-GMA/QAn+TMDI/TEGMA 40 wt.%/40 wt.%/20 wt.% (40(QAn+TMDI)_p_) also had higher WS and SL compared to the Bis-GMA/UDMA/TEGMA 40 wt.%/40 wt.%/20 wt.% (40(UDMA)_p_) reference, which had the WS = 11.71 µg/mm^3^ and SL = 1.12 µg/mm^3^ [46]. Copolymers with the highest antibacterial activity and the best physico-mechanical parameters, i.e., 40(QAn+TMDI)_p_s with n = 8, 10, and 12, had the WS from 35.54 to 68.27 µg/mm^3^, and SL from 5.15 to 5.22 µg/mm^3^. This corresponded to 3.04 to 5.83 times higher WS values and on average 4.6 times higher SL values than 40(UDMA)_p_ (regarding that the WS decreased, while SL increased with the increase in the length of the N-alkyl substituent) [46]. In addition, compared to the WS and SL values specified in the ISO 4049 (WS = 40 µg/mm^3^, and SL = 7.5 µg/mm^3^ [47]), only 40(QA12+TMDI)_p_ had lower WS, while all 40(QAn+TMDI)_p_s had the SL within the recommended limit. The developed further Bis-GMA/QAn+TMXDI/UDMA/TEGDMA 40 wt.%/20 wt.%/20 wt.%/20 wt.%: 20(QA10+TMXDI)_p_ and 20(QA12+TMXDI)_p_ copolymers had a WS of 10.43 and 10.35 µg/mm^3^, and SL of 2.18 and 2.46 µg/mm^3^, respectively. These values were still higher than those determined for the 40(UDMA)_p_ reference [44] but notably lower than those specified in the ISO 4049 standard [47].

This article presents six novel QAUDMAs having cores derived from: isophorone diisocyanate (IPDI), 4,4′-methylenedicyclohexyl diisocyanate (CHMDI), and 4,4′-diphenylmethane diisocyanate (MDI), and QA groups substituted with N-octyl (QA8+IPDI, QA8+CHMDI, QA8+MDI) and N-decyl substituents (QA10+IPDI, QA10+CHMDI, QA10+MDI) (Figure 1). These monomers were obtained from cycloaliphatic and aromatic diisocyanates because we hypothesized that their copolymerization with dental dimethacrylates could lower the water sorption and solubility. We checked this hypothesis by investigating the WS and SL of six experimental copolymers consisting of Bis-GMA 40 wt.%, QAUDMA (QAn+IPDI, QAn+CHMDI, and QAn+MDI, where n = 8 and 10) 40 wt.%, and TEGDMA 20 wt.%. We discussed the results obtained concerning the WS and SL values identified in the ISO 4049 standard as an upper limit [47], and those of the reference copolymer consisted of Bis-GMA 40 wt.%, UDMA 40 wt.%, and TEGDMA 20 wt.% (40(UDMA)_p_).

These findings can deepen the understanding of the water behavior of copolymers containing QAUDMA repeating units and also inspire further research into their potential applications in dental materials science.

## 2. Results

### 2.1. Synthesis

In this research, six novel QAUDMA monomers were successively synthesized: QA8+IPDI, QA10+IPDI, QA8+CHMDI, QA10+CHMDI, QA8+MDI, and QA10+MDI (Figure 1).

**Figure 2:**
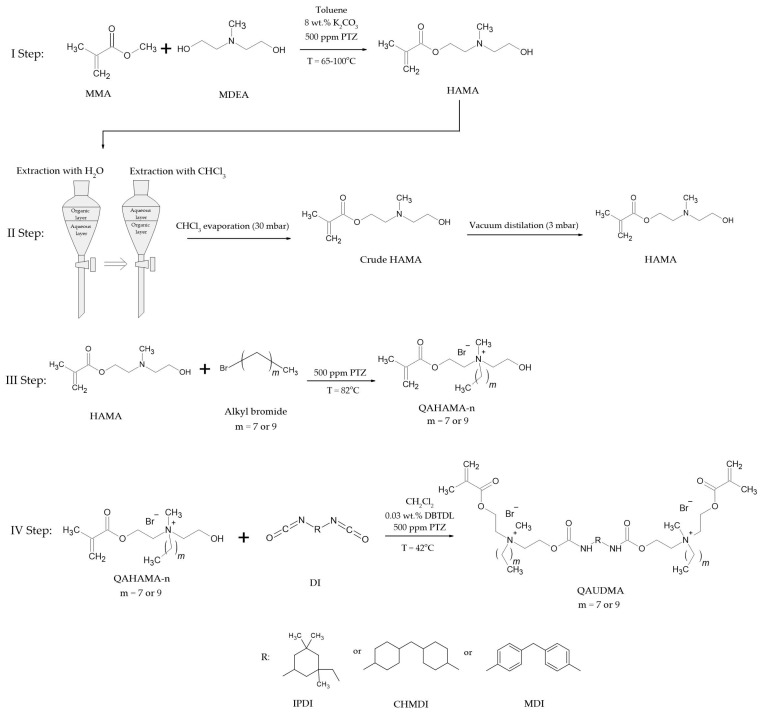
The synthesis route of QAUDMA monomers utilizing MDEA, MMA, OB, or DB (n = 8, and 10, respectively) and IPDI, CHMDI, or MDI as substrates.

The synthesis route was based on a procedure developed for the synthesis of QAn+TMDI monomers (Figure 2) [42]. The first stage involved the transesterification of methyl methacrylate (MMA) and N-methyldiethanolamine (MDEA), catalyzed by potassium carbonate (K2CO3), which results in the formation of HAMA. In the second stage, a series of operations was carried out to purify the raw product. This begins with the extraction of the toluene reaction mixture with water, where HAMA and unreacted MDEA moved into the aqueous layer. Next, the aqueous layer was extracted with chloroform, which captured HAMA. After chloroform evaporation, the crude HAMA was subjected to vacuum distillation, free from contaminants such as the catalyst, solvents, or phenothiazine. The third stage involved the N-alkylation of HAMA with octyl bromide (OB) and decyl bromide (DB), leading to the formation of QAHAMA-n compounds: specifically, QAHAMA-8 and QAHAMA-10. In the fourth stage, an addition reaction was carried out between QAHAMA-n and diisocyanate (IPDI, CHMDI, and MDI), catalyzed by dibutyltin dilaurate (DBTDL). This reaction resulted in the formation of QAUDMA compounds: QA8+IPDI, QA10+IPDI, QA8+CHMDI, QA10+CHMDI, QA8+MDI, and QA10+MDI. The purity of all intermediate and final products was verified using 1H NMR, 13C NMR, and FTIR spectroscopic analysis.

### 2.2. Spectroscopic Analysis of Monomers

Final products as well as intermediate products were subjected to spectroscopic analysis utilizing ^1^H NMR, ^13^C NMR, and FTIR. ^1^H NMR and ^13^C NMR spectra of substrates and intermediate products can be found in the Supplementary Materials (Figures S1–S10, Tables S1–S20).

#### 2.2.1. ^1^H NMR

**Figure 3:**
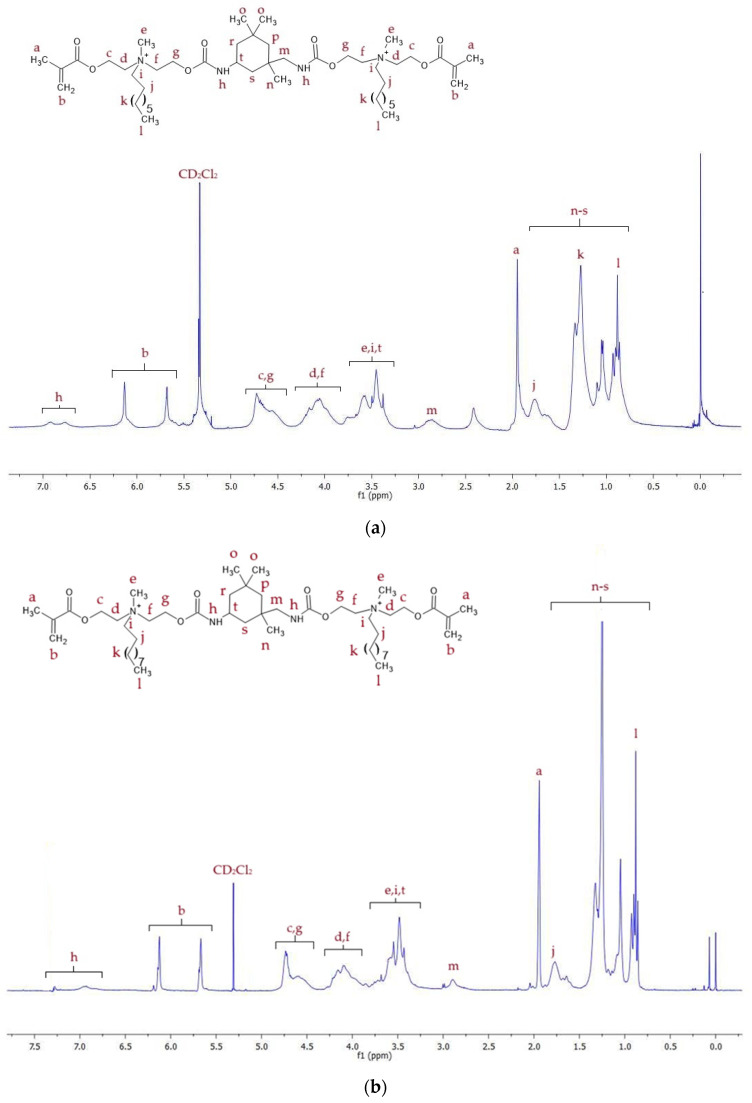
^1^H NMR spectra of: (**a**) QA8+IPDI and (**b**) QA10+IPDI monomers.

^1^H NMR spectra of QA8+IPDI and QA10+IPDI are shown in Figure 3, whereas signal description is shown in Table S21.

**Figure 4:**
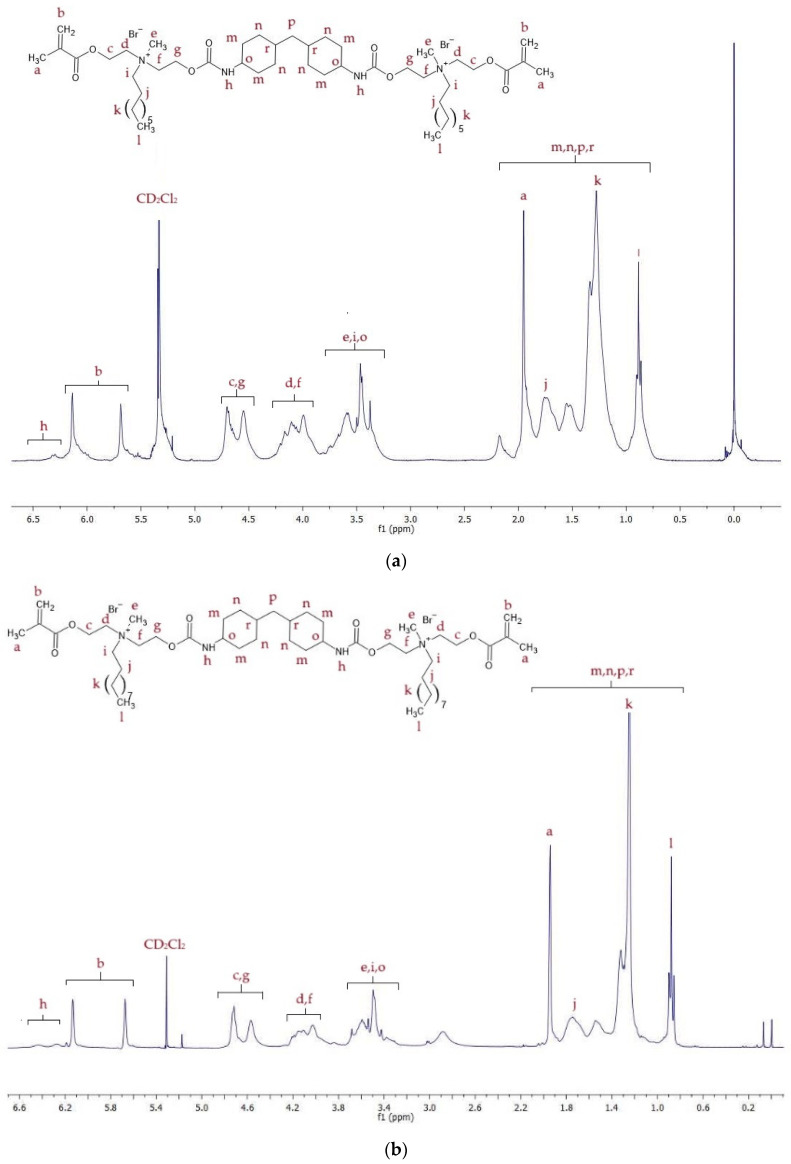
^1^H NMR spectra of (**a**) QA8+CHMDI and (**b**) QA10+CHMDI monomers.

^1^H NMR spectra of QA8+CHMDI and QA10+CHMDI are shown in Figure 4, whereas signal description is shown in Table S22.

**Figure 5:**
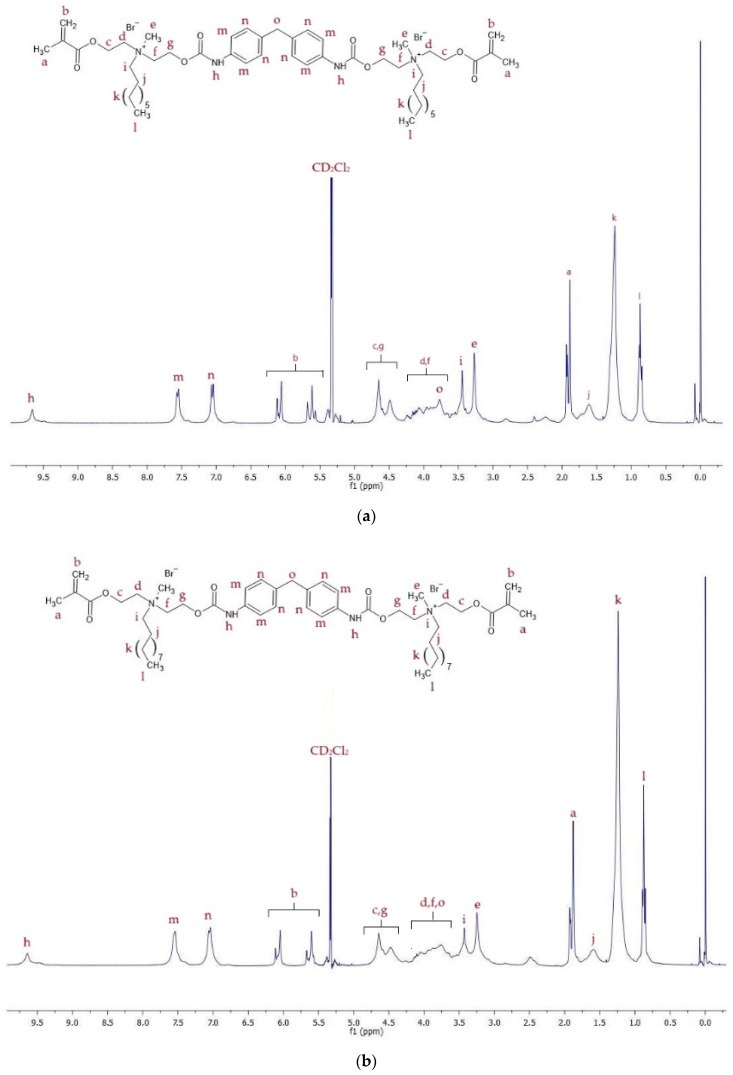
^1^H NMR spectra of (**a**) QA8+MDI and (**b**) QA10+MDI monomers.

^1^H NMR spectra of QA8+MDI and QA10+MDI are shown in Figure 5, whereas signal description is shown in Table S23.

#### 2.2.2. ^13^C NMR

**Figure 6:**
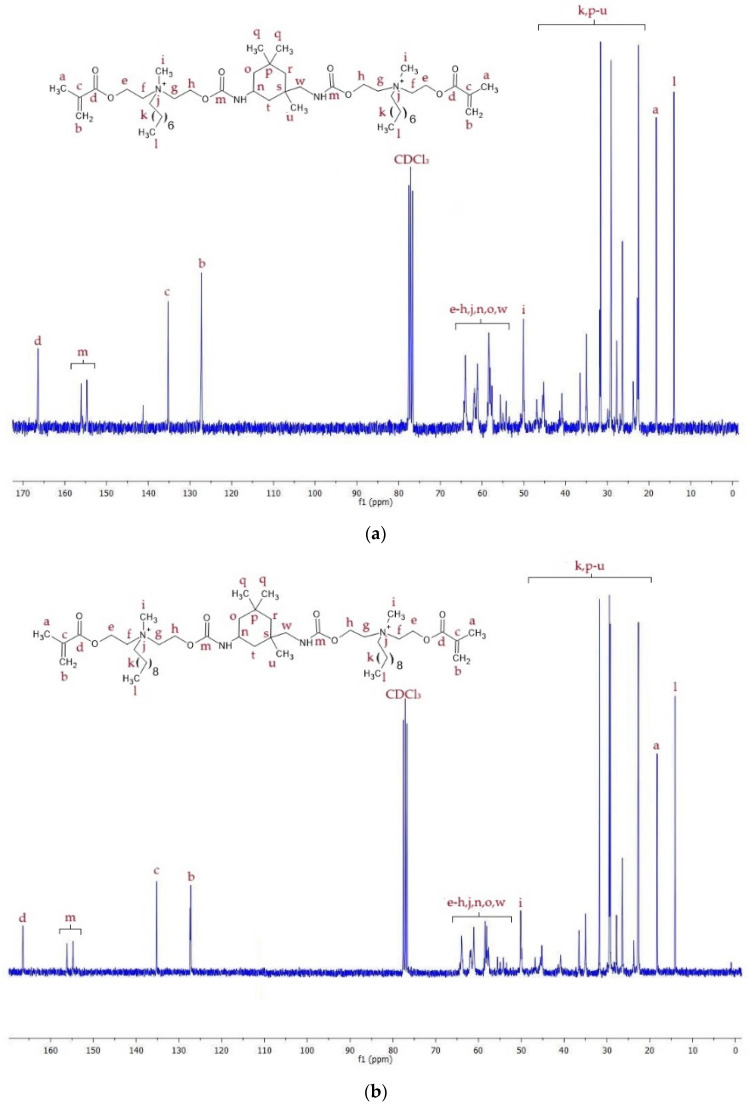
^13^C NMR spectra of (**a**) QA8+IPDI and (**b**) QA10+IPDI monomers.

^13^C NMR spectra of QA8+IPDI and QA10+IPDI are shown in Figure 6, whereas signal description is shown in Table S24.

**Figure 7:**
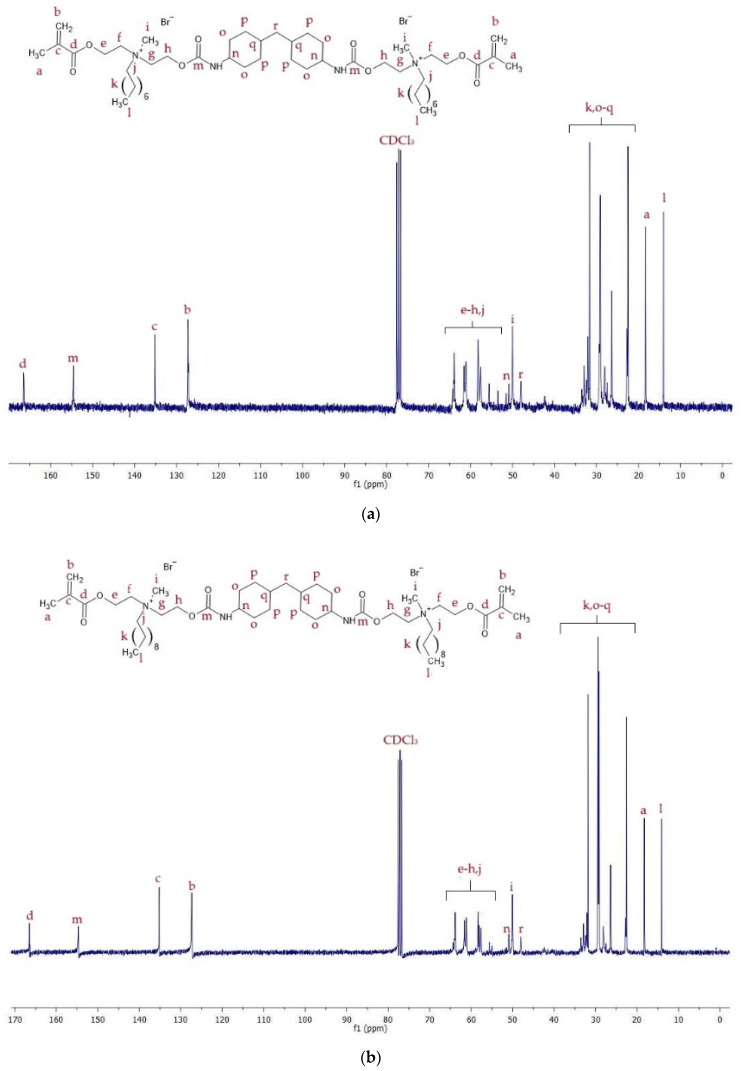
^13^C NMR spectra of (**a**) QA8+CHMDI and (**b**) QA10+CHMDI monomers.

^13^C NMR spectra of QA8+CHMDI and QA10+CHMDI are shown in Figure 7, whereas signal description is shown in Table S25.

**Figure 8:**
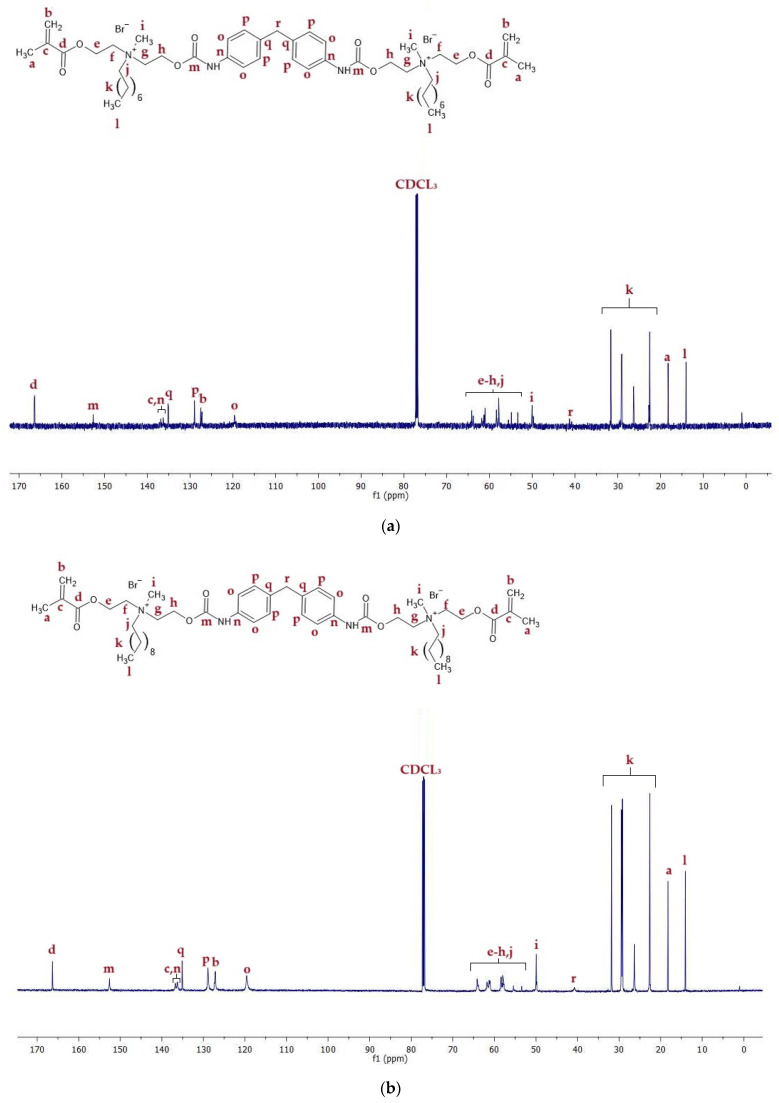
^13^C NMR spectra of (**a**) QA8+MDI and (**b**) QA10+MDI monomers.

^13^C NMR spectra of QA8+MDI and QA10+MDI are shown in Figure 8, whereas signal description is shown in Table S26.

#### 2.2.3. FTIR

**Figure 9:**
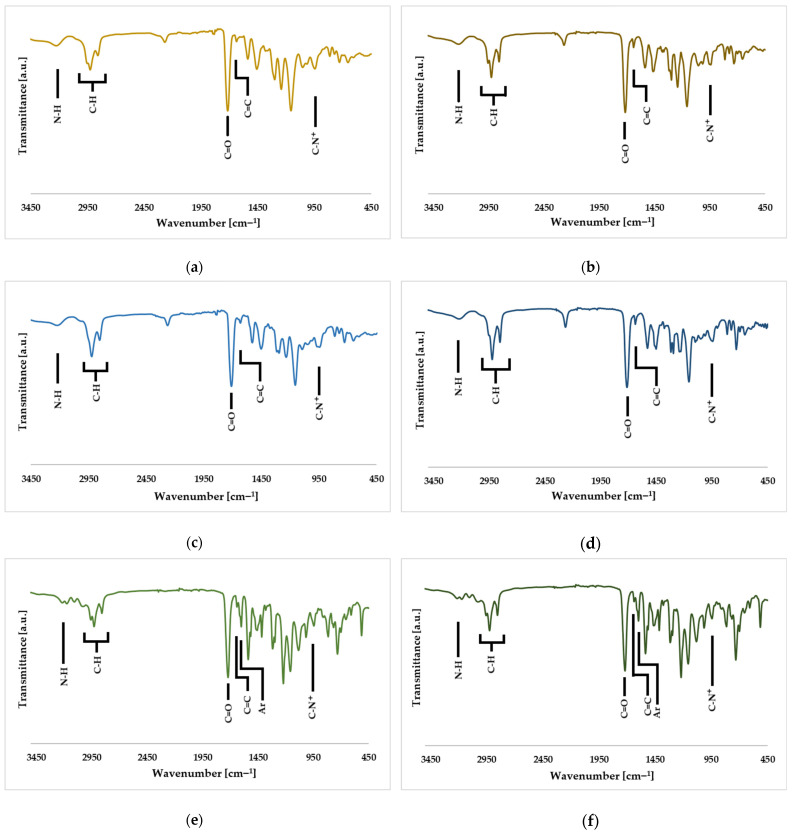
FTIR spectra of (**a**) QA8+IPDI, (**b**) QA10+IPDI, (**c**) QA8+CHMDI, (**d**) QA10+CHMDI, (**e**) QA8+MDI, and (**f**) QA10+MDI monomers.

FTIR spectra of QA8+IPDI, QA10+IPDI, QA8+CHMDI, QA10+CHMDI, QA8+MDI, and QA10+MDI monomers are shown in Figure 9, whereas signal description is shown in Tables S27–S29.

### 2.3. Monomers

The novel QAUDMA monomers QA8+IPDI, QA10+IPDI, QA8+CHMDI, QA10+CHMDI, QA8+MDI and QA10+MDI were characterized for the following properties: (i) molecular weight (MW), (ii) concentration of double bonds (x_DB_), (iii) concentration of urethane bonds (x_UB_), (iv) refractive index (RI), (v) density (d_m_) (Table 1).

The molecular weight (MW) of synthesized QAUDMAs ranged from 983 to 1079 g/mol. It increased with the MWs of the N-alkyl bromide and diisocyanate, according to the following order: IPDI < MDI < CHMDI. The concentration of double bonds (x_DB_), which equaled the concentration of urethane bonds (x_UB_), ranged from 1.85 to 2.03 mol/kg and decreased with the increasing MW of QAUDMA. In the case of the UDMA reference monomer, x_DB_ = x_UB_ was 4.25. The average MW of QAUDMAs was higher by 54% than the MW of UDMA, and the x_DB_ = x_UB_ of UDMA was higher by 54% than the average x_DB_ = x_UB_ of QAUDMAs.

The refractive index (RI) of QAUDMAs ranged from 1.5130 to 1.5595, and it decreased with the increasing length of the N-alkyl substituent. Additionally, the RI values increased in the following order of DIs: IPDI < CHMDI < MDI. All the results for the RI of QAUDMAs were statistically significant (*p* < 0.05). The average RI value for QAUDMAs was higher than that of UDMA by 4.5%.

The density (d_m_) of synthesized QAUDMAs ranged from 1.18 to 1.52 g/cm^3^. QAn+IPDIs and QAn+CHMDIs had lower d_m_ values than QAn+MDIs and these differences were statistically significant, *p* < 0.05. The differences between the d_m_ of QA8+IPDI and QA8+CHMDI, and QA10+IPDI and QA10+CHMDI were statistically insignificant (*p* ≥ 0.05). The d_m_ decreased with the length of the N-alkyl substituent for QAn+IPDIs and QAn+CHMDIs, whereas it increased for QAn+MDIs (these decreases in the d_m_ values were statistically significant, *p* < 0.05). The d_m_ values for QAUDMAs were higher than the d_m_ value of UDMA from 8 to 39%.

### 2.4. Copolymers

The synthesized QAUDMAs were combined with commercial DMAs in the following weight fractions: QAUDMA 40 wt.%, Bis-GMA 40 wt.%, and TEGDMA 20 wt.%, and cured with the CQ/DMAEMA photoinitiating system. For comparison, a reference composition was made, which consisted of UDMA 40 wt.%, Bis-GMA 40 wt.%, and TEGDMA 20 wt.%. The resulting copolymers—40(QA8+IPDI)_p_, 40(QA10+IPDI)_p_, 40(QA8+CHMDI)_p_, 40(QA10+CHMDI)_p_, 40(QA8+MDI)_p_, and 40(QA10+MDI)_p_—were characterized for water sorption (WS) and water solubility (SL) (Table 2).

The results presented in Table 2 show that the WS of 40(QAUDMA)_p_s ranged from 11.63 to 14.00 μg/mm^3^. Its values decreased with the increasing length of the N-alkyl substituent. Additionally, the WS values decreased in the following order of DIs: IPDI > CHMDI > MDI. The differences in the WS values of 40(QA8+IPDI)_p_, 40(QA10+IPDI)_p_, and 40(QA8+CHMDI)_p_ were statistically insignificant, as well as the WS values of 40(QA10+CHMDI)_p_, 40(QA8+MDI)_p_, and 40(QA10+MDI)_p_ (*p* ≥ 0.05). The WS values of all 40(QAUDMA)_p_s were 2.2 to 2.6 times higher than that of 40(UDMA)_p_, and these differences were statistically significant (*p* < 0.05). Considering the limit value indicated in the ISO 4049 standard, 40 μg/mm^3^ [47], the WS values of 40(QAUDMA)_p_s were from 29 to 35% of this limit. The WS of 40(UDMA)_p_ was 13% of this limit for comparison.

The water solubility (SL) of 40(QAUDMA)_p_s ranged from 2.62 to 7.68 μg/mm^3^. No visible effect of the N-alkyl substituent length on SL was observed for 40(QAn+IPDI)_p_s, and 40(QAn+CHMDI)_p_s (the differences in the SL values were statistically insignificant, *p* ≥ 0.05, except the 40(QA10+IPDI)_p_, and 40(QA10+CHMDI)_p_ couple, *p* < 0.05). The SL values increased in the following order of DIs: IPDI ≈ CHMDI < MDI. The SL values for all 40(QAUDMA)_p_s were from 2.28 to 6.74 times higher than that of 40(UDMA)_p_, and these differences were statistically significant (*p* < 0.05). Taking into account the limit value indicated in the ISO 4049 standard, which is 7.5 μg/mm^3^ [47], the SL values for 40(QAn+IPDI)_p_s and 40(QAn+CHMDI)_p_s were from 36 to 43% of this limit. The SL value of 40(QA10+MDI)_p_ was 90% of this limit, while the SL value of 40(QA8+MDI)_p_ exceeded this limit by 5.68%. The SL of 40(UDMA)_p_ was 16% of this limit for comparison.

## 3. Discussion

This research presents six QAUDMA monomer structures, which include three core variants derived from the IPDI, CHMDI, and MDI diisocyanates and two wing variants derived from QAHAMA-8 and QAHAMA-10 (Figure 1). These specific DIs represent an innovative approach, as they were not previously employed in synthesizing QAUDMA monomers. The choice of QAHAMA-8 and QAHAMA-10 was dictated by our earlier findings, which demonstrated that 40(QAn+TMDI)_p_s (where n = 8, 10, and 12) exhibited high antibacterial activity alongside good mechanical properties. We observed that these properties improved as the length of the N-alkyl substituents decreased [43]. However, a significant drawback of the 40(QAn+TMDI)_p_s was the high water sorption, which increased as the length of the N-alkyl substituent decreased [46]. Neither 40(QA8+TMDI)_p_ nor 40(QA10+TMDI)_p_ met the water sorption limit of less than 40 µg/mm^3^, as specified in standard ISO 4049. Notably, 40(QA12+TMDI)_p_ was the first copolymer of that series, which had a WS value compatible with the criteria established in ISO 4049 [47]. In this study, to effectively reduce water sorption we developed six QAUDMAs by replacing aliphatic TMDI core with IPDI, CHMDI, and MDI and we limited the number of n-alkyl bromides used to the OB and DB, resulting in the incorporation of N-octyl and N-decyl substituents.

The synthesis route for the novel QAUDMAs was successfully adopted from the literature (Figure 2) [42]. In the first step, HAMA was synthesized with a methacrylate group on one end, a hydroxyl group on the other, and a tertiary amino group in the center. In the second step, HAMA was transformed into QAHAMA-n (specifically QAHAMA-8 and QAHAMA-10) through the quaternization of the tertiary amino group via the Menschutkin reaction, using OB and DB, respectively. In the third step, QAHAMA-n was subjected to an addition reaction with three diisocyanates: IPDI, CHMDI, and MDI. This reaction resulted in six compounds: QA8+IPDI, QA10+IPDI, QA8+CHMDI, QA10+CHMDI, QA8+MDI, and QA10+MDI. The ^1^H NMR, ^13^C NMR, and FTIR spectroscopic analyses confirmed the success of obtaining novel QAUDMAs with the designed chemical structure.

The ^1^H NMR spectra displayed proton signals corresponding to all chemical groups in the QAUDMAs structure (Figure 3, Figure 4 and Figure 5, Tables S21–S23). The formation of two carbamate groups was confirmed by observing the -NH-(C=O)-O- signals at 6.27–6.93 ppm. The remaining signals corresponded to: (i) two methacrylate groups (-CH_3_ at 1.92–1.94 ppm and =CH_2_ at 5.61–5.67 and 6.06–6.12 ppm); (ii) four MDEA-derived methylene groups (-O-CH_2_-CH_2_-N^+^- at 3.80–4.30 ppm and -O-CH_2_-CH_2_-N^+^- at 4.30–4.75 ppm); (iii) two methyl groups neighboring to two quaternary nitrogen atoms (-N^+^-CH_3_ at 3.27–3.70 ppm); (iv) two N-alkyl substituents, including -N^+^-CH_2_- at 3.30–3.70 ppm, -N^+^-CH_2_-CH_2_- at 1.50–1.80 ppm, -(CH_2_)_5 (or 7)_-CH_3_ at 1.00–1.40 ppm, and -CH_3_ at 0.85–0.88 ppm; (v) groups of the IPDI core (-CH_2_-NH- at 2.89 ppm, >C(CH_3_)_2_ and -CH_2_- at 0.86–1.90 ppm, and >CH-NH- at 3.30–3.70 ppm); (vi) groups of the CHMDI core (-CH_2_- and -CH-C< at 0.85–1.94 ppm, and -NH-CH< at 3.30–3.70 ppm); and (vii) groups of the MDI core (-CH-_(Ar)_ at 7.00–7.20 and 7.40–7.70 ppm, and -CH_2_- at 3.77 ppm).

The ^13^C NMR spectra displayed signals of carbon atoms corresponding to all chemical groups present in the QAUDMAs structure (Figure 6, Figure 7 and Figure 8, Tables S24–S26). The formation of carbamate groups was confirmed by observing -NH-(C=O)-O- signals at 155–157 ppm. The remaining signals corresponded to: (i) the methacrylate group (-CH_3_ at 19 ppm, =CH_2_ at 126–128 ppm, CH_2_=C< at 135–138 ppm, and -COO- at 166 ppm); (ii) the MDEA-derived methylene groups (-O- CH_2_- CH_2_-N^+^-: series of peaks at 55–66 ppm); (iii) a methyl group neighboring to a quaternary nitrogen atom (-N^+^-CH_3_ at 49–50 ppm); (iv) an N-alkyl substituent (-CH_2_-N^+^- at 55–66 ppm, -(CH_2_)_6(or 8)_- (series of peaks at 23–46 ppm), and -CH_3_ at 14 ppm); (v) groups of the IPDI core (-CH_2_-NH-, and >CH-NH- at 58–66 ppm, >C(CH_3_)_2_, >C(CH_2_)(CH_3_)-, and -CH_2_- at 23–46 ppm); (vi) groups of the CHMDI core (>CH-CH_2_-CH< at 49 ppm), (vii) -CH_2_- and -CH< at 23–35 ppm, and -NH-CH< at 51 ppm; and (viii) groups of the MDI core (-C_(Ar)_-NH- at 138 ppm, -CH_(Ar)_- at 120–129 ppm, -C_(Ar)_- at 137 ppm, and -C_(Ar)_-CH_2_-C_(Ar)_- at 42 ppm).

The FTIR spectra revealed bond vibrations adequate to the chemical structure of synthesized QAUDMAs: (i) the N-H stretching vibrations at 3215 cm⁻^1^ and N-H in-plane bending vibrations at 1529–1536 cm⁻^1^, both associated with the -NH-CO-O- carbamate group; (ii) C=O stretching vibrations at 1714–1718 cm⁻^1^, associated with both -NH-CO-O- carbamate and -CO-O- methacrylate groups; (iii) two C-O stretching bands, one at 1044–1077 cm⁻^1^ and the other at 1008–1010 cm⁻^1^, also associated with both -NH-CO-O- carbamate and -CO-O- methacrylate groups; (iv) C-N⁺ vibrations at 941–943 cm⁻^1^; (v) C-H stretching vibrations at 2848–3066 cm⁻^1^, and scissoring vibrations at 1455–1460 cm⁻^1^, in aliphatic -CH_3_ and -CH_2_- groups; (vi) C=C stretching vibrations at 1628–1638 cm⁻^1^; and (vii) aromatic C=C stretching vibrations at 1613 cm⁻^1^, found only in the QAn+MDI spectra (Figure 9, Tables S27–S29).

Each QAUDMA had two double bonds and two urethane bonds, so x_DB_ equaled the concentration of urethane bonds (x_UB_). As the N-alkyl substituent lengthened, the MW increased while x_DB_ = x_UB_ decreased. The MWs of QAUDMAs can also be ranked by diisocyanate (DI) in this increasing order: IPDI < MDI < CHMDI. This corresponds to decreasing x_DB_ = x_UB_ (Table 1). It is worth noting that higher concentrations of double and urethane bonds indicate a polymer network with higher crosslink density. Double bonds lead to chemical crosslinking, while urethane bonds are related to physical crosslinking [48]. It suggests that the longer the N-alkyl substituent in the QAUDMA, the lower the theoretical crosslink density of the resulting polymer network, and the QAn+CHMDI-derived networks will theoretically be the tightest.

The QAUDMAs were also tested for the RI, which is the optical property responsible for the high translucency of DCRMs and determines the esthetic quality of dental fillings. To achieve DCRMs with optimal translucency, the RI values of the inorganic fillers and polymer matrix should be similar. Ideal RI values for the fillers are 1.47 to 1.52, ensuring they match the RI of the dimethacrylate matrix. Any mismatch can lead to light refraction and reflection at the filler–matrix interfaces [49]. The RI values of the QAUDMAs, being the subject of this study, ranged from 1.5130 to 1.5595 (Table 1), which demonstrated that they were usually within the recommended RI range for dental dimethacrylates, approaching the upper limit of this range. Only QAn+MDIs exhibited higher RIs than the upper threshold.

Detailed analysis of the RI values by the QAUDMA chemical structure showed that they were influenced by both the N-alkyl substituent length and diisocyanate core. RI values were higher for the N-octyl substituent than for the N-decyl substituent, which indicates that a higher number of carbon atoms in the alkyl chain attached to the nitrogen cation could increase the RI. Furthermore, the following order of increasing RI values for diisocyanates emerged: IPDI < CHMDI < MDI. This finding indicates that incorporating benzene rings in the QAUDMA structure could increase the RI. Similar results were reported by Bauer et al., who tested the RIs of hexamethylene diisocyanate- and MDI-derived polyurethanes, finding that the latter had a higher RI [50].

QAUDMAs had densities ranging from 1.18 to 1.52 g/cm^3^ (Table 1). When compared to the densities of dental dimethacrylates—1.15 g/cm^3^ for Bis-GMA, 1.09 g/cm^3^ for UDMA, and 1.07 g/cm^3^ for TEGDMA [42]—the QAUDMAs exhibited higher densities. The presence of the aromatic rings significantly increased the monomer density compared to these containing cycloaliphatic rings. Additionally, the length of the N-alkyl substituent affected density in various ways. The N-octyl substituent resulted in higher d_m_ values than N-decyl for QAn+IPDIs and QAn+CHMDIs. Zec et al. observed a similar effect for imidazolium-based bromide ionic liquids. Their densities decreased with increasing alkyl chain length on the imidazolium cation [51]. On the contrary, QA8+MDI had a lower density than QA10+MDI. This is consistent with the general relationship between the number of carbon atoms and the density of alkanes, which increases with the increase in the n-alkane MW [52].

The obtained QAUDMA monomers were utilized to modify a standard DCRM dental dimethacrylate formulation. This formulation initially consisted of 40 wt.% UDMA, 40 wt.% Bis-GMA, and 20 wt.% TEGDMA, creating a standard DCRM matrix. The strategic decision was to replace the UDMA entirely with one of the QAUDMAs to reduce water sorption (WS) and solubility (SL) of the resulting copolymers in comparison to the corresponding 40(QAn+TMDI)_p_s and to meet the WS and SL values indicated in the ISO 4049 standard [47] as maximum for dental materials. As a result, the newly formulated copolymers comprised QAUDMA 40 wt.%, Bis-GMA 40 wt.%, and TEGDMA 20 wt.% (40(QAUDMA)_p_s).

Polymers containing quaternary ammonium groups are always challenging in dental material science, primarily due to their high water sorption. This issue arises from the strong water affinity of the QA groups. It can deteriorate the performance characteristics of DCRMs. The high WS of the composite matrix negatively affects the DCRMs’ mechanical and dimensional stability by reducing the mechanical strength, stiffness, and hardness and causing hygroscopic expansion [31,53,54]. While hygroscopic expansion can be advantageous on a small scale, helping to compensate for polymerization shrinkage and potentially eliminating marginal gaps, excessive water absorption can lead to over-compensation, which can create internal expansion stress, ultimately jeopardizing the integrity of the restored tooth [55].

The WS values of 40(QAUDMA)_p_s were from 11.63 to 14.00 μg/mm^3^ (Table 2). These values, when compared to the 40 μg/mm^3^ WS value specified in ISO 4049, were found to be within 29% to 35% of that threshold. Moreover, a comparison of the measured WS values revealed a significant improvement in water sorption compared to previously reported data for the corresponding 40(QAn+TMDI)_p_s. Specifically, the 40(QA8+TMDI)_p_ had a WS of 68.27 μg/mm^3^, while the 40(QA10+TMDI)_p_ had a WS of 48.42 μg/mm^3^, being 1.71 and 1.21 times higher than the standard of 40 μg/mm^3^ [47].

The N-alkyl substituent length and the DI-derived core in the QAUDMA units influenced the WS of 40(QAUDMA)_p_s. The longer the N-alkyl substituent, the lower the WS. The hydrophobic character of the hydrocarbon chain associated with its increased length probably restricted the diffusion of water molecules into the crosslinked copolymer structure [52]. Secondly, the WS values of 40(QAUDMA)_p_s decreased according to the following order of diisocyanates: TMDI > IPDI > CHMDI > MDI. This order reflects the chemical structure of the DIs: fully aliphatic > cycloaliphatic asymmetrical > cycloaliphatic symmetrical > aromatic symmetrical. Interestingly, 40(QAn+MDI)_p_s exhibited the lowest WS values, while one could expect 40(QAn+CHMDI)_p_s to have the lowest WS. The lowest x_DB_ = x_UB_ values were seen in 40(QAn+CHMDI)_p_s, indicating the formation of the tightest networks, and a more significant hydrophobic effect of the cyclohexane moiety compared to the aromatic ring could support this hypothesis [56]. The monomer density can partially explain these differences. Since QAn+MDIs had the highest d_m_ values among the investigated QAUDMAs due to the planar conformation of the benzyne ring in MDI, it likely makes it more difficult for water molecules to penetrate the structure of 40(QAn+MDI)_p_s.

Another crucial factor in the effective functioning of composite dental filling is water solubility (SL). It must be sufficiently low to ensure the restoration maintains stable dimensions and mechanical properties. Additionally, preventing the leaching of low-molecular-weight compounds from the DRCM matrix is essential, as this helps avoid potential mouth tissue irritation and toxic effects on the human body [24,25].

The investigated 40(QAUDMA)_p_s exhibited an SL from 2.62 to 7.68 μg/mm^3^ (Table 2). When comparing these SL values to the value of 7.5 μg/mm^3^ cited in ISO 4049 standard as a maximum SL limit, it is evident that most of the obtained copolymers operated within 36% to 90% of this threshold. However, the SL values of 40(QA8+MDI)_p_ exceed this limit by 5%. This finding demonstrated the diversity in water behavior within the 40(QAUDMA)_p_s. In addition to the solubility limits, the analysis of the measured SL values indicated a significant improvement in some cases compared to previously reported data for 40(QA8+TMDI)_p_ with an SL of 5.15 μg/mm^3^ and 40(QA10+TMDI)_p_ with an SL of 5.18 μg/mm^3^ [46]. These SL values were approximately 69% of the 7.5 μg/mm^3^ limit. The copolymers with QAUDMA units derived from cycloaliphatic DIs: 40(QA8+IPDI)_p_, 40(QA10+IPDI)_p_, 40(QA8+CHMDI)_p_, and 40(QA10+CHMDI)_p_, showed improvement in their solubility characteristics, having SL values, respectively, of 36, 41, 41, and 43% of the 7.5 μg/mm^3^ limit.

The SL values of 40(QAUDMA)_p_s can be ranked by diisocyanate (DI) in this decreasing order: MDI > TMDI > CHMDI > IPDI. Notably, the 40(QAn+IPDI)_p_s and 40(QAn+CHMDI)_p_s had the lowest SL. It might be related to the fact that the saturated cyclohexane ring increases the hydrophobicity of the 40(QAUDMA)_p_ structure more than the aromatic ring [56]. Therefore, this can make it harder for QAn+IPDI and QAn+CHMDI sol fractions to be leached out of the crosslinked 40(QAUDMA)_p_ structure.

In conclusion, the investigated 40(QAUDMA)_p_s exhibited satisfactory water behavior, significantly improved compared to 40(QAn+TMDI)_p_s. The 40(QAn+MDI)_p_s had the lowest WS. However, they had the highest SL, with QA8+MDI exceeding the recommended limit. The most promising candidates for further research appeared to be 40(QAn+IPDI)_p_s and 40(QAn+CHMDI)_p_s, i.e., copolymers containing cycloaliphatic moieties, as they exhibited the lowest WS and SL. It can be expected that after adding the filler, the WS and SL will decrease [55].

This article can serve as a preliminary study that initiates a series of investigations on the physicochemical, mechanical, and biological properties of 40(QAUDMA)_p_s. We expect that findings from these studies will facilitate the selection of polymers with the most promising characteristics, ultimately leading to the development of innovative antibacterial dental composite restorative materials, having the potential to reduce the incidence of secondary caries.

## 4. Materials and Methods

### 4.1. Materials

Methyl methacrylate (MMA), N-methyldiethanolamine (MDEA), octyl bromide (OB), and decyl bromide (DB) were purchased from Acros Organics (Geel, Belgium). Isophorone diisocyanate (IPDI), dicyclohexylmethane 4,4′-diisocyanate (CHMDI), 1,1′-methylenebis(4-isocyanatobenzene) (MDI), phenothiazine (PTZ), camphorquinone (CQ), N,N-dimethylaminoethyl methacrylate (DMAEMA), and tetramethylsilane (TMS) were purchased from Sigma-Aldrich (St. Louis, MO, USA). Dibutyltin dilaurate (DBTDL) was purchased from Fluka (Charlotte, NC, USA). Deuterated trichloromethane (CDCl_3_) and deuterated dichloromethane (CD_2_Cl_2_) were purchased from Deutero GMBH (Kastellaun, Germany). Potassium carbonate (K_2_CO_3_) and magnesium sulfate (MgSO_4_) were purchased from Chempur (Piekary Śląskie, Poland). Toluene, trichloromethane (CHCl_3_), and dichloromethane (CH_2_Cl_2_) were purchased from Stanlab (Lublin, Poland).

### 4.2. Chemical Syntheses

#### 4.2.1. N,N-(2-Hydroxyethyl)methylaminoethyl Methacrylate (HAMA)

MMA and MDEA were subjected to the transesterification reaction utilizing K_2_CO_3_ as a catalyst and PTZ as a free radical polymerization inhibitor. The reaction was carried out in toluene solution. The quantities of reagents and other chemicals used in the reaction are listed in Table 3.

The compounds listed in Table 3 were placed in a 1000 mL single-neck round-bottom flask, which was equipped with a Vigreux column and distillation head. The reaction mixture was brought to a boil, and the azeotropic mixture of MMA, methanol, and toluene was continuously collected. The reaction was conducted until the temperature at the column head reached 100 °C. Once the reaction was completed, K_2_CO_3_ was filtered, and the filtrate was washed three times with distilled water in a 1:2 volume ratio. The aqueous fractions were combined and extracted three times with CHCl_3_ in a 1:3 volume ratio. The CHCl_3_ fractions were also combined, and a residual water was dried overnight using MgSO_4_. CHCl_3_ was subsequently evaporated utilizing a rotary evaporator IKA RV 10 digital with the heating bath IKA HB 10 digital (IKA Werke, Staufen im Breisgau, Germany). and oil vacuum pump TANKER 230 (Rocker, Kaohsiung, Tajwan) under reduced pressure (30 mbar). The resulting raw product was subjected to vacuum distillation at 3 mbar utilizing an oil vacuum pump TANKER 230 (Rocker, Kaohsiung, Tajwan), collecting the boiling fraction between 110 °C and 130 °C. This process yielded HAMA at 14%.

#### 4.2.2. 2-(Methacryloyloxy)ethyl-2-Hydroxyethylmethylalkylammonium Bromides (QAHAMA-n)

In a three-necked round-bottom flask, HAMA and alkyl bromide (either OB or DB) were subjected to the N-alkylation reaction, along with PTZ (a free radical polymerization inhibitor) (Table 4). The flask was equipped with a mechanical stirrer, a reflux condenser, and a thermometer. The reaction was carried out at 82 °C for 90 h. The yield for 2-(methacryloyloxy)ethyl-2-hydroxyethylmethyloctylammonium bromide (QAHAMA-8) and 2-(methacryloyloxy)ethyl-2-decylhydroxyethylmethylammonium bromide (QAHAMA-10) was 100%.

#### 4.2.3. Quaternary Ammonium Urethane-Dimethacrylates (QAUDMA)

A 50% solution of QAHAMA-n (the addition reaction reagent) and DBTDL (the catalyst) in a CH_2_Cl_2_ was introduced into a three-necked round-bottom flask, which was equipped with a reflux condenser, thermometer, and dropping funnel. The contents of the flask were heated to the boiling point of CH_2_Cl_2_ (approximately 42 °C). Next, a 50% solution of the diisocyanate (second addition reaction reagent) in CH_2_Cl_2_ was added dropwise over a period of 1.5 h. Following this, the reaction mixture was maintained at the boiling point and allowed to continue for an additional 5 h (Table 5).

After the synthesis was complete, the solvent was evaporated a rotary evaporator IKA RV 10 digital with the heating bath IKA HB 10 digital (IKA Werke, Staufen im Breisgau, Germany). and oil vacuum pump TANKER 230 (Rocker, Kaohsiung, Tajwan) under reduced pressure (first at 30 mbar, then at 3 mbar). The final products remaining in the flask appeared as a light-yellow, viscous liquid. The flask containing the product was then placed in a laboratory dryer (SLW 53 STD, Wodzisław Śląski, Poland) and heated at 42 °C for 24 h. A 100% process yield was achieved for each QAUDMA monomer.

### 4.3. Curing Procedure and Sample Preparation

Six experimental monomer compositions were subjected to photopolymerization, which resulted in the following copolymers:

- 40(QA8+IPDI)_p_: QA8+IPDI 40 wt.%, Bis-GMA 40 wt.%, and TEGDMA 20 wt.%
- 40(QA10+IPDI)_p_:QA10+IPDI 40 wt.%, Bis-GMA 40 wt.%, and TEGDMA 20 wt.%
- 40(QA8+CHMDI)_p_: QA8+CHMDI 40 wt.%, Bis-GMA 40 wt.%, and TEGDMA 20 wt.%
- 40(QA10+CHMDI)_p_: QA10+CHMDI 40 wt.%, Bis-GMA 40 wt.%, and TEGDMA 20 wt.%
- 40(QA8+MDI)_p_: QA8+MDI 40 wt.%, Bis-GMA 40 wt.%, and TEGDMA 20 wt.%
- 40(QA10+MDI)_p_: QA10+MDI 40 wt.%, Bis-GMA 40 wt.%, and TEGDMA 20 wt.%.

The reference copolymer, 40(UDMA)_p_, consisted of: UDMA 40 wt.%, Bis-GMA 40 wt.%, and TEGDMA 20 wt.%.

Experimental monomer compositions, along with a reference monomer composition, were prepared utilizing mechanical stirring at a temperature of 50 °C. The initiating system comprised CQ 0.4 wt.% and DMAEMA 1 wt.%, which was incorporated into the homogeneous monomer mixtures. Stirring continued at 50 °C until the complete dissolution of CQ was achieved. The resultant compositions were poured into molds, covered with PET foil, and subjected to irradiation using a UV-Vis lamp (Ultra Vitalux 300, Osram, Munich, Germany) at room temperature for one hour. The lamp emitted radiation within the spectral range of 280 to 780 nm and provided a radiation exitance of 2400 mW/cm^2^.

### 4.4. Nuclear Magnetic Resonance Spectroscopy (NMR)

The NMR spectra of substrates, intermediate products, and monomers were obtained using 300 MHz and 600 MHz spectrometers (UNITY/INOVA, Varian, Palo Alto, CA, USA). For the ^1^H NMR spectra, 256 scans were collected, while for the ^13^C NMR spectra, 40,000 scans were performed. The ^1^H NMR spectra were analyzed as solutions in CD_2_Cl_2_, whereas the ^13^C NMR spectra were analyzed in CDCl_3_. TMS was used as the internal standard in each experiment.

### 4.5. Fourier Transform Infrared Spectroscopy (FTIR)

The FTIR spectra were collected at a resolution of 1 cm^−1^ using the Spectrum Two spectrometer (Perkin-Elmer, Waltham, MA, USA) with 128 scans. Monomers were subjected to analysis as a thin layer positioned between two KBr pellets.

### 4.6. Refractive Index

The refractive index (RI) was determined with a DR 6100T (Krüss Optronic, Hamburg, Germany) digital refractometer. The procedure was carried out in accordance with the ISO 489:2022 standard [57].

### 4.7. Density

The density of QAUDMA monomers was determined by the pycnometer method, following the instructions in the ISO 1675:2022 standard [58]. A 1 mL pycnometer acc. to Gay-Lussac made of borosilicate glass 3.3 (Paul Marienfeld GmbH & Co. KG, Lauda-Königshofen, Germany) was used in the experiments.

### 4.8. Water Sorption and Solubility

The water sorption (WS) and solubility (SL) of copolymers were evaluated using disk-like specimens. These specimens had dimensions of 15 mm diameter and 1.0 mm thickness and weighed 0.3 g. The tests were conducted in accordance with the guidelines of the ISO 4049:2019 standard [47]. An analytical balance (XP Balance, Mettler Toledo, Greifensee, Switzerland) was used for the experiments. Initially, the specimens were dried to a constant weight (m_0_) in a laboratory dryer (SLW 53 STD, POL-EKO, Wodzisław Śląski, Poland). Following the drying process, the specimens were immersed in deionized water and stored at room temperature for 7 days. After this period, the specimens were removed from the water, dried with blotting paper, and weighed (m_1_). The specimens were then dried again until a constant weight (m_2_) was achieved. The values of WS and SL were calculated using the following equations:

WSμgmm3=m1−m0V,

SLμgmm3=m0−m2V,

The variables in the equation are defined as follows:

m_0_—the initial mass of the dried samples

m_1_—the mass of the swollen samples after water storage

m_2_—the mass of the dried samples after water storage

V—the initial volume of dried samples

### 4.9. Statistical Analysis

The non-parametric Wilcoxon test was conducted using Statistica 13.3 software (TIBCO Software Inc., Palo Alto, CA, USA) with a significance level (*p*) of 0.05. Statistical significance was assessed for five samples. Results were presented as the average value (AV) and its corresponding standard deviation (SD).

## Figures and Tables

**Table 1:** The molecular weight (MW), concentration of double bonds (x_DB_), concentration of urethane bonds (x_UB_), refractive index (RI), and density (d_m_) of synthesized QAUDMA monomers and UDMA reference monomer. All results for the RI were statistically significant (*p* < 0.05). The results for the d_m_ usually were statistically significant (*p* < 0.05), except the results for couples highlighted with the lowercase letters in the superscripts, which were statistically insignificant (*p* ≥ 0.05).

Monomer	MW (g/mol)	x_DB_ = x_UB_ (mol/kg)	RI	d_m_ (g/cm^3^)	
			AV ^1^	AV	SD
Experimental monomers					
QA8+IPDI	983	2.03	1.5211	1.24 ^a^	0.04
QA10+IPDI	1039	1.92	1.5130	1.18 ^b^	0.02
QA8+CHMDI	1023	1.96	1.5234	1.25 ^a^	0.01
QA10+CHMDI	1079	1.85	1.5205	1.19 ^b^	0.02
QA8+MDI	1011	1.98	1.5595	1.32	0.02
QA10+MDI	1067	1.87	1.5535	1.52	0.01
Reference monomer					
UDMA	470	4.25	1.4614	1.09	0.01

^1^ The SD for the RI values was 0.0001.

**Table 2:** The water sorption (WS) and water solubility (SL) of the 40(QAUDMA)_p_ experimental copolymers and 40(UDMA)_p_ reference copolymer. The statistically significant results (*p* < 0.05) for the WS and SL are highlighted with the lowercase letters in the superscripts.

Copolymer	WS (μg/mm^3^)		SL (μg/mm^3^)	
	AV	SD	AV	SD
Experimental copolymers				
40(QA8+IPDI)_p_	14.00 ^a,g,h,i,^	1.61	2.70 ^a,g,h^	0.29
40(QA10+IPDI)_p_	13.18 ^b,j,k,l^	1.02	2.62 ^b,i,j,k,l^	0.26
40(QA8+CHMDI)_p_	13.75 ^c,m,n,o^	1.32	2.94 ^c,i,m,n^	0.21
40(QA10+CHMDI)_p_	11.79 ^d,g,j,m^	0.14	3.08 ^d,j,o,p^	0.20
40(QA8+MDI)_p_	11.67 ^e,h,k,n^	0.32	7.68 ^e,g,k,m,o,r^	0.48
40(QA10+MDI)_p_	11.63 ^f,i,l,o^	0.47	6.61 ^f,h,l,n,p,r^	0.53
Reference copolymer				
40(UDMA)_p_	5.85 ^a,b,c,d,e,f^	0.49	1.13 ^a,b,c,d,e,f^	0.04

**Table 3:** The quantities of chemical substances used in the reaction for obtaining HAMA.

Substance	Mass (g)	Moles (mol)
MMA	100.12	1.00
MDEA	79.85	0.67
K_2_CO_3_	14.40	^1^
PTZ	0.05	^1^
Toluene	346	^1^

^1^ The number of moles was insignificant.

**Table 4:** The quantities of chemical substances used in the reaction for obtaining QAHAMA-n (n = 8 and 10).

Substance	Mass (g)	Moles (mol)
HAMA	20.00	0.107
OB	20.66	0.107
DB	23.67	0.107
PTZ	0.01	^1^

^1^ The number of moles was insignificant.

**Table 5:** The names of QAUDMA monomers and quantities of chemical substances used in the reaction of their synthesis.

QAUDMA	Substance	Mass (g)	Moles (mol)
QA8+IPDI	QAHAMA-8	20.34	0.053
	IPDI	5.94	0.027
	DBTDL	0.008	^1^
QA10+IPDI	QAHAMA-10	20.34	0.050
	IPDI	5.54	0.025
	DBTDL	0.01	^1^
QA8+CHMDI	QAHAMA-8	22.82	0.060
	CHMDI	7.97	0.030
	DBTDL	0.01	^1^
QA10+CHMDI	QAHAMA-10	22.82	0.056
	CHMDI	7.33	0.028
	DBTDL	0.01	^1^
QA8+MDI	QAHAMA-8	23.50	0.062
	MDI	7.73	0.031
	DBTDL	0.01	^1^
QA10+MDI	QAHAMA-10	23.50	0.058
	MDI	7.20	0.029
	DBTDL	0.01	^1^

^1^ The number of moles was insignificant.

## Data Availability

Data are contained within the article and Supplementary Materials.

## Supplementary Materials

The following supporting information can be downloaded at: https://www.mdpi.com/article/10.3390/molecules30040769/s1, Figure S1: The NMR Spectra of methyl methacrylate: (a) ^1^H NMR (b) ^13^C NMR; Figure S2: The NMR Spectra of N-methyldiethanolamine: (a) ^1^H NMR (b) ^13^C NMR; Figure S3: The NMR spectra of N,N-(2-hydroxyethyl)methylaminoethyl methacrylate: (a) ^1^H NMR (b) ^13^C NMR; Figure S4: The NMR spectra of 1-bromooctane: (a) ^1^H NMR (b) ^13^C NMR; Figure S5: The NMR spectra of 1-bromodecane: (a) ^1^H NMR (b) ^13^C NMR; Figure S6: The NMR spectra of 2-(methacryloyloxy)ethyl-2-hydroxyethylmethyloctylammonium bromide: (a) ^1^H NMR (b) ^13^C NMR; Figure S7: The NMR spectra of 2-(methacryloyloxy)ethyl-2-decylhydroxyethylmethylammonium bromide: (a) ^1^H NMR (b) ^13^C NMR; Figure S8: The NMR spectra of isophorone diisocyanate: (a) ^1^H NMR (b) ^13^C NMR; Figure S9: The NMR spectra of dicyclohexylmethane 4,4′-diisocyanate: (a) ^1^H NMR (b) ^13^C NMR; Figure S10: The NMR Spectra of 1,1′-methylenebis(4-isocyanatobenzene): (a) ^1^H NMR (b) ^13^C NMR. Table S1: ^1^H NMR signals of methyl methacrylate; Table S2: ^13^C NMR signals of methyl methacrylate; Table S3: ^1^H NMR signals of N-methyldiethanolamine; Table S4: ^13^C NMR signals of N-methyldiethanolamine; Table S5: ^1^H NMR signals of N,N-(2-hydroxyethyl)methylaminoethyl methacrylate; Table S6: ^13^C NMR signals of N,N-(2-hydroxyethyl)methylaminoethyl methacrylate; Table S7: ^1^H NMR signals of 1-bromooctane; Table S8: ^13^C NMR signals of 1-bromooctane; Table S9: ^1^H NMR signals of 1-bromodecane; Table S10: ^13^C NMR signals of 1-bromodecane; Table S11: ^1^H NMR signals of 2-(methacryloyloxy)ethyl-2-hydroxyethylmethyloctylammonium bromide; Table S12: ^13^C NMR signals of 2-(methacryloyloxy)ethyl-2-hydroxyethylmethyloctylammonium bromide; Table S13: ^1^H NMR signals of 2-(methacryloyloxy)ethyl-2-decylhydroxyethylmethylammonium bromide; Table S14: ^13^C NMR signals of 2-(methacryloyloxy)ethyl-2-decylhydroxyethylmethylammonium bromide; Table S15: ^1^H NMR signals of isophorone diisocyanate; Table S16: ^13^C NMR signals of isophorone diisocyanate; Table S17: ^1^H NMR signals of dicyclohexylmethane 4,4’-diisocyanate; Table S18: ^13^C NMR signals of dicyclohexylmethane 4,4’-diisocyanate; Table S19: ^1^H NMR signals of 1,1′-methylenebis(4-isocyanatobenzene); Table S20: ^13^C NMR signals of 1,1′-methylenebis(4-isocyanatobenzene); Table S21: ^1^H NMR signals of QA8+IPDI and QA10+IPDI monomers; Table S22: ^1^H NMR signals of QA8+CHMDI and QA10+CHMDI monomers; Table S23: ^1^H NMR signals of QA8+MDI and QA10+MDI monomers; Table S24: ^13^C NMR signals of QA8+IPDI and QA10+IPDI monomers; Table S25: ^13^C NMR signals of QA8+CHMDI and QA10+CHMDI monomers; Table S26: ^13^C NMR signals of QA8+MDI and QA10+MDI monomers; Table S27: The interpretation of FTIR spectra of QA8+IPDI and QA10+IPDI monomers; Table S28: The interpretation of FTIR spectra of QA8+CHMDI and QA10+CHMDI monomers; Table S29: The interpretation of FTIR spectra of QA8+MDI and QA10+MDI monomers.
