# Supplementary material for: Novel Quaternary Ammonium Urethane-Dimethacrylates for Copolymers with Low Water Sorption and Solubility
Source: Molecules. 2025 Feb 7;30(4):769. doi: 10.3390/molecules30040769 (PMC11858556; doi:10.3390/molecules30040769)
Supplement: Supplementary file 1 [file molecules-30-00769-s001.zip › molecules-3428640-supplementary.pdf]

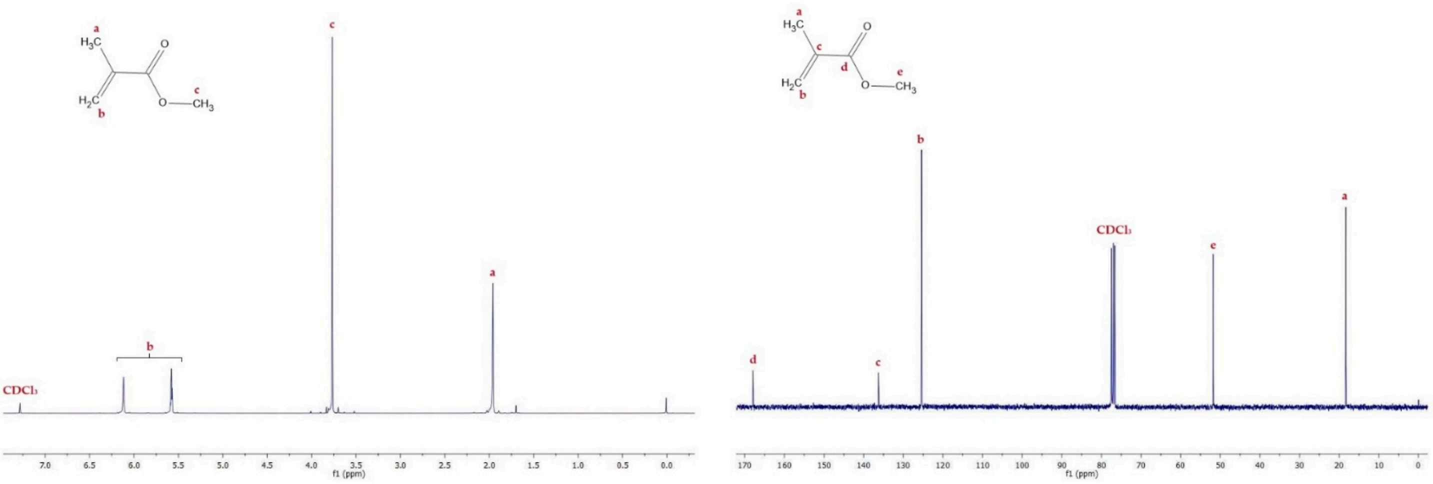

a) b)

Figure S1. The NMR Spectra of methyl methacrylate: a) ^{1}H NMR b) ^{13}C NMR.

Table S1. ^{1}H NMR signals of methyl methacrylate.

Signal Symbol	Hydrogen Atom	Multiplicity	Number of Protons	Chemical Shift (ppm)
a	$C\underline{H}_3$-C=	s	3	1.96
b	=$C\underline{H}_2$	2m	2	5.58 and 6.12
c	-O-$C\underline{H}_3$	s	3	3.77

Table S2. ^{13}C NMR signals of methyl methacrylate.

Signal Symbol	Carbon Atom	Chemical Shift (ppm)
a	$\underline{C}H_3$-C=	18
b	$\underline{C}H_2$=	125
c	CH_2=$\underline{C}$<	139
d	-$\underline{C}$OO-	165
e	-O-$\underline{C}H_3$	52

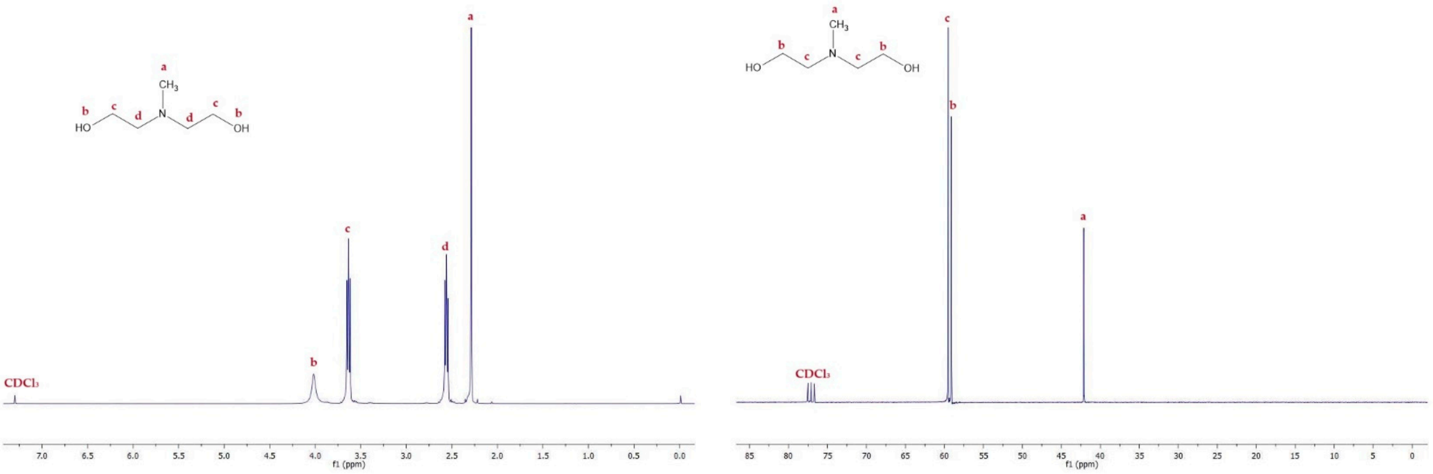

a) b)

Figure S2. The NMR Spectra of N-methyldiethanolamine: a) ^{1}H NMR b) ^{13}C NMR.

Table S3. ^{1}H NMR signals of N-methyldiethanolamine.

Signal Symbol	Hydrogen Atom	Multiplicity	Number of Protons	Chemical Shift (ppm)
a	$C\underline{H}_3$-N-	s	3	2.29
b	$\underline{H}$O-	s	2	4.00
c	HO-$C\underline{H}_2$-	t	4	3.50-3.70
d	-$C\underline{H}_2$-N-	t	4	2.40-2.65

Table S4. ^{13}C NMR signals of N-methyldiethanolamine.

Signal Symbol	Carbon Atom	Chemical Shift (ppm)
a	$\underline{C}H_3$-N-	42
b	HO-$\underline{C}H_2$-	59
c	-$\underline{C}H_2$-N-	60

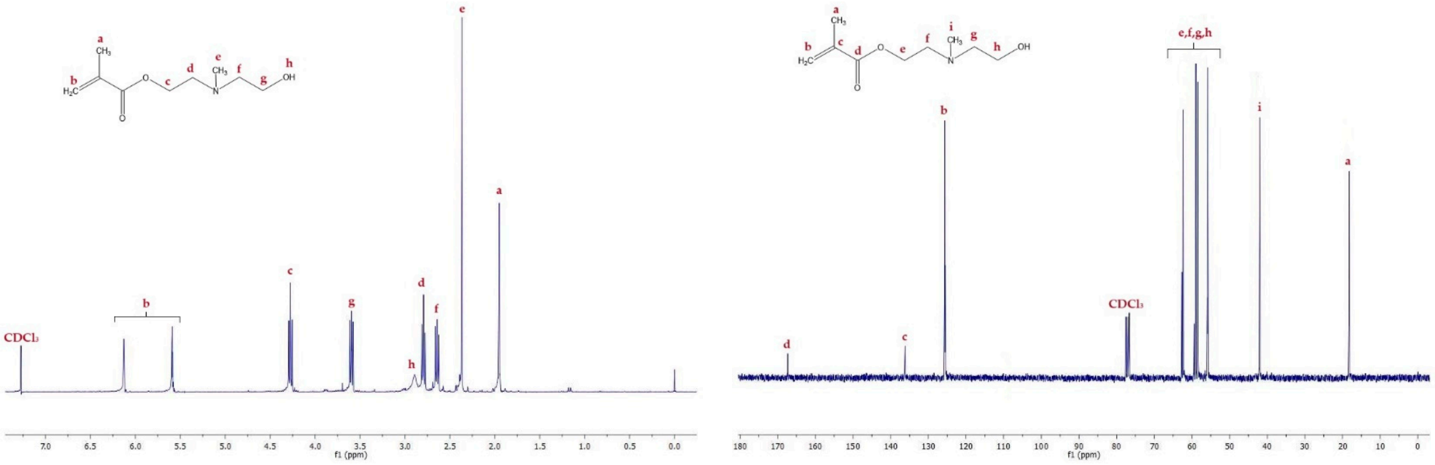

a) b)

Figure S3. The NMR spectra of N,N-(2-hydroxyethyl)methylaminoethyl methacrylate: a) ^{1}H NMR b) ^{13}C NMR.

Table S5. ^{1}H NMR signals of N,N-(2-hydroxyethyl)methylaminoethyl methacrylate.

Signal Symbol	Hydrogen Atom	Multiplicity	Number of Protons	Chemical Shift (ppm)
a	$C\underline{H_3}$-C=	s	3	1.95
b	=$C\underline{H_2}$	2m	2	5.60 and 6.13
c	-$C\underline{H_2}$-	t	2	4.22-4.34
d	-$C\underline{H_2}$-	t	2	2.75-2.84
e	$C\underline{H_3}$-	s	3	2.37
f	-$C\underline{H_2}$-	t	2	2.59-2.70
g	-$C\underline{H_2}$-	t	2	3.40-3.65
h	$\underline{H}$O-	s	1	2.86-2.97

Table S6. ^{13}C NMR signals of N,N-(2-hydroxyethyl)methylaminoethyl methacrylate.

Signal Symbol	Carbon Atom	Chemical Shift (ppm)
a	$\underline{C}H_3$-C=	18
b	$\underline{C}H_2$=	126
c	CH_2=$\underline{C}$<	136
d	-$\underline{C}$OO-	167
e-h	-$\underline{C}H_2$-	54-63
i	$\underline{C}H_3$-N	42

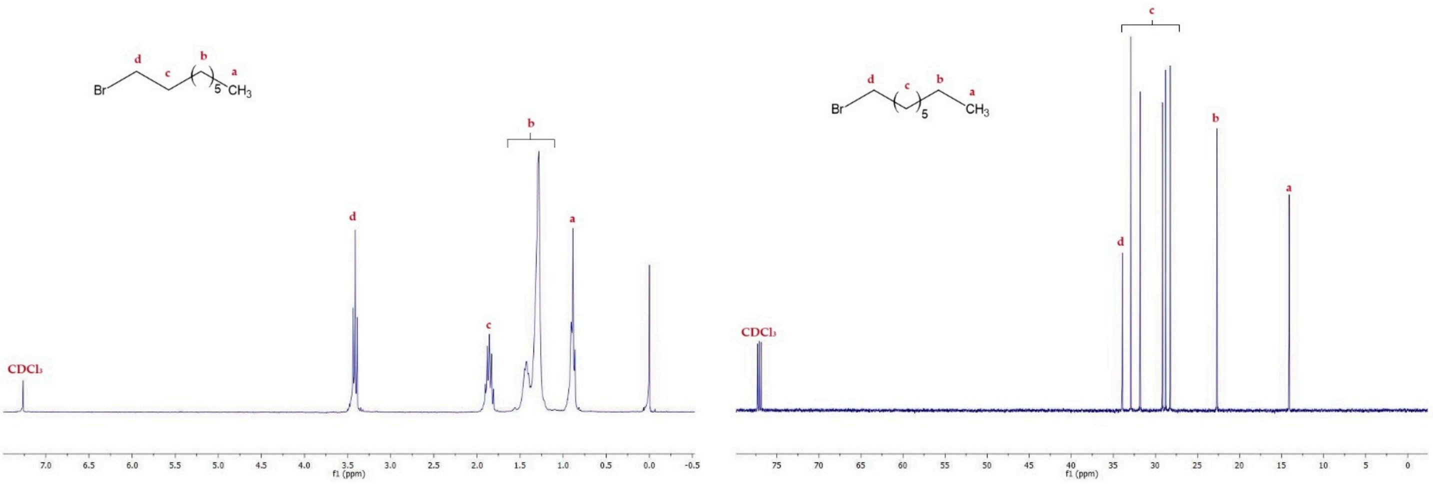

a) b)

Figure S4. The NMR spectra of 1-bromooctane: a) ^{1}H NMR b) ^{13}C NMR.

Table S7. ^{1}H NMR signals of 1-bromooctane.

Signal Symbol	Hydrogen Atom	Multiplicity	Number of Protons	Chemical Shift (ppm)
a	$C\underline{H}_3$-	m	3	0.80-1.00
b	-$C\underline{H}_2$-	m	10	1.16-1.54
c	-$C\underline{H}_2$-	m	2	1.75-1.96
d	-$C\underline{H}_2$-Br	m	2	3.29-3.54

Table S8. ^{13}C NMR signals of 1-bromooctane.

Signal Symbol	Carbon Atom	Chemical Shift (ppm)
a	$\underline{C}H_3$-	14
b	-$\underline{C}H_2$-	23
c	-$\underline{C}H_2$-	28-33
d	-$\underline{C}H_2$-Br	34

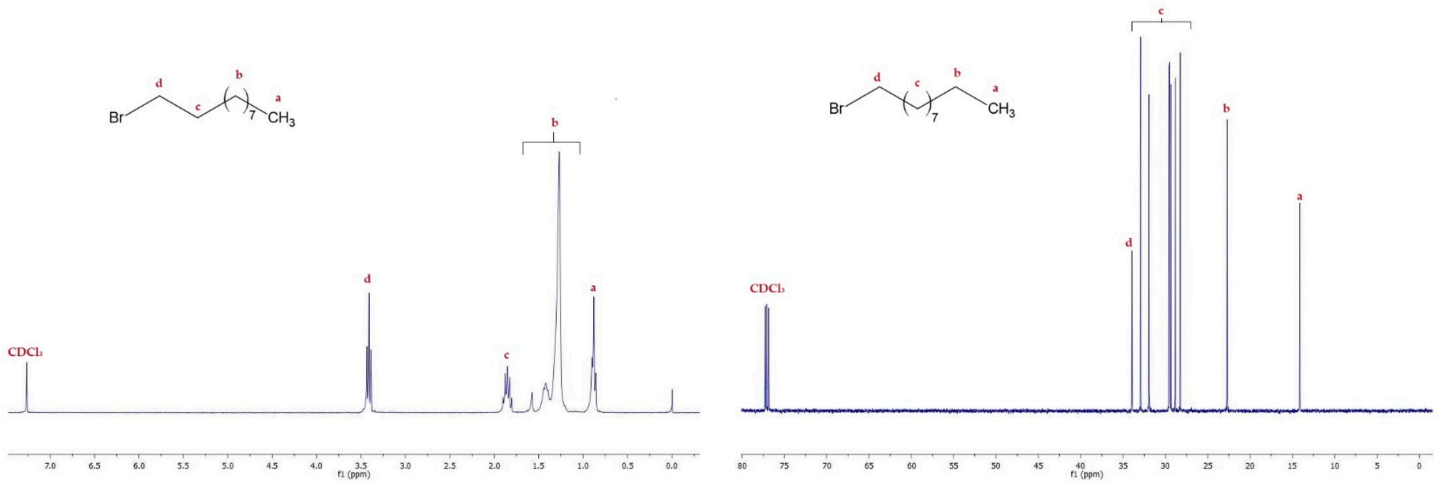

a) b)

Figure S5. The NMR spectra of 1-bromodecane: a) ^{1}H NMR b) ^{13}C NMR.

Table S9. ^{1}H NMR signals of 1-bromodecane.

Signal Symbol	Hydrogen Atom	Multiplicity	Number of Protons	Chemical Shift (ppm)
a	C$\underline{H}_3$-	m	3	0.80-0.98
b	-C$\underline{H}_2$-	m	14	1.15-1.63
c	-C$\underline{H}_2$-	m	2	1.78-1.95
d	-C$\underline{H}_2$-Br	m	2	3.34-3.51

Table S10. ^{13}C NMR signals of 1-bromodecane.

Signal Symbol	Carbon Atom	Chemical Shift (ppm)
a	$\underline{C}H_3$-	14
b	-$\underline{C}H_2$-	23
c	-$\underline{C}H_2$-	28-33
d	-$\underline{C}H_2$-Br	34

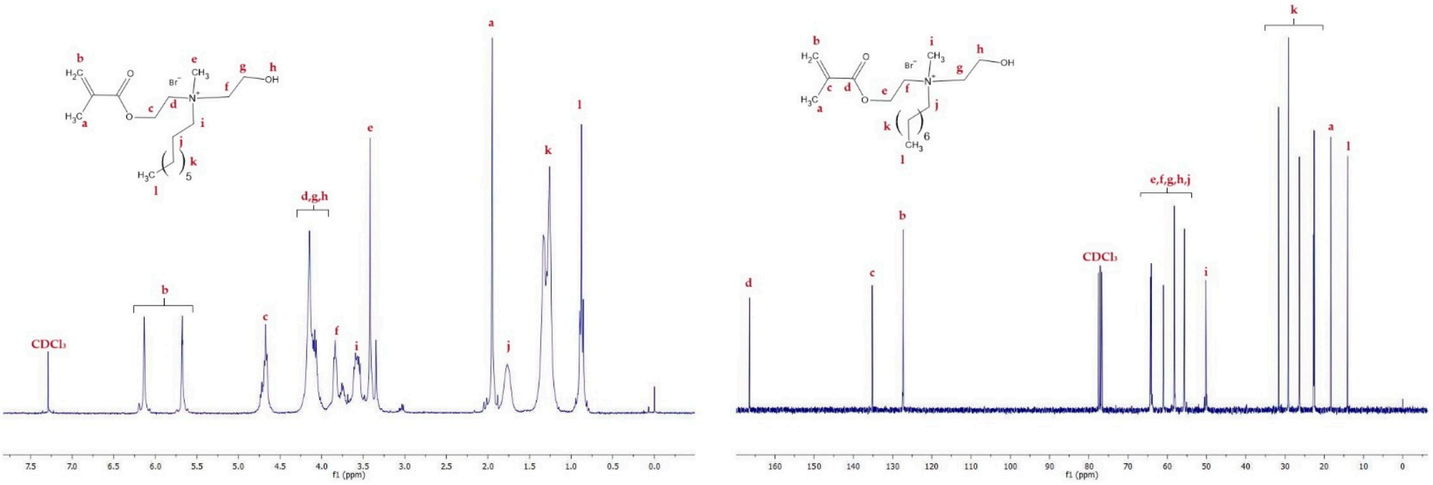

a) b)

Figure S6. The NMR spectra of 2-(methacryloyloxy)ethyl-2-hydroxyethylmethyloctylammonium bromide: a) ^{1}H NMR b) ^{13}C NMR.

Table S11. ^{1}H NMR signals of 2-(methacryloyloxy)ethyl-2-hydroxyethylmethyloctylammonium bromide.

Signal Symbol	Hydrogen Atom	Multiplicity	Number of Protons	Chemical Shift (ppm)
a	$C\underline{H}_3$-C=	s	3	1.95
b	=$C\underline{H}_2$	2m	2	5.68 and 6.13
c	-$C\underline{H}_2$-	m	2	4.58-4.77
d	-$C\underline{H}_2$-	m	2	3.97-4.27
e	$C\underline{H}_3$-N^+	s	3	3.27-3.47
f	-$C\underline{H}_2$-	m	2	3.69-3.90
g	-$C\underline{H}_2$-	m	2	3.97-4.27
h	$\underline{H}$O-	m	1	3.97-4.27
i	-$C\underline{H}_2$-	m	2	3.52-3.65
j	-$C\underline{H}_2$-	s	2	1.68-1.85
k	-$C\underline{H}_2$-	m	10	1.13-1.45
l	$C\underline{H}_3$-	m	3	0.88

Table S12. ^{13}C NMR signals of 2-(methacryloyloxy)ethyl-2-hydroxyethylmethyloctylammonium bromide.

Signal Symbol	Carbon Atom	Chemical Shift (ppm)
a	$\underline{C}H_3$-C=	18
b	$\underline{C}H_2$=	127
c	CH_2=$\underline{C}$<	135
d	-$\underline{C}$OO-	166
e-h	-$\underline{C}H_2$-	53-65
i	$\underline{C}H_3$-N^+	50
j	-$\underline{C}H_2$-	53-65
k	-$\underline{C}H_2$-	21-33
l	$\underline{C}H_3$-	14

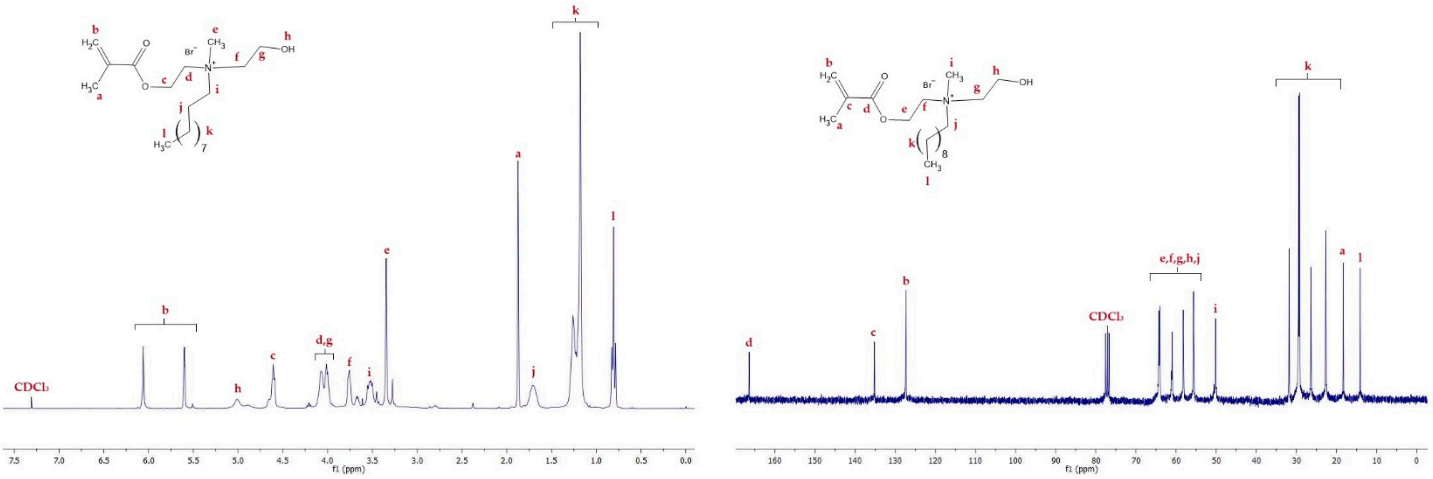

a) b)

Figure S7. The NMR spectra of 2-(methacryloyloxy)ethyl-2-decylhydroxyethylmethylammonium bromide: a) ^{1}H NMR b) ^{13}C NMR.

Table S13. ^{1}H NMR signals of 2-(methacryloyloxy)ethyl-2-decylhydroxyethylmethylammonium bromide.

Signal Symbol	Hydrogen Atom	Multiplicity	Number of Protons	Chemical Shift (ppm)
a	CH_3-C=	s	3	1.87
b	=CH_2	2m	2	5.61 and 6.01
c	-CH_2-	m	2	4.51-4.72
d	-CH_2-	m	2	3.94-4.16
e	CH_3-N^+	s	3	3.22-3.40
f	-CH_2-	m	2	3.61-3.82
g	-CH_2-	m	2	3.94-4.16
h	HO-	m	1	4.83-5.01
i	-CH_2-	m	2	3.43-3.60
j	-CH_2-	s	2	1.61-1.81
k	-CH_2-	m	14	1.06-1.37
l	CH_3-	m	3	0.81

Table S14. ^{13}C NMR signals of 2-(methacryloyloxy)ethyl-2-decylhydroxyethylmethylammonium bromide.

Signal Symbol	Carbon Atom	Chemical Shift (ppm)
a	CH_3-C=	18
b	CH_2=	127
c	CH_2=C<	135
d	-COO-	166
e-h	-CH_2-	54-66
i	CH_3-N^+	50
j	-CH_2-	54-66
k	-CH_2-	21-33
l	CH_3-	14

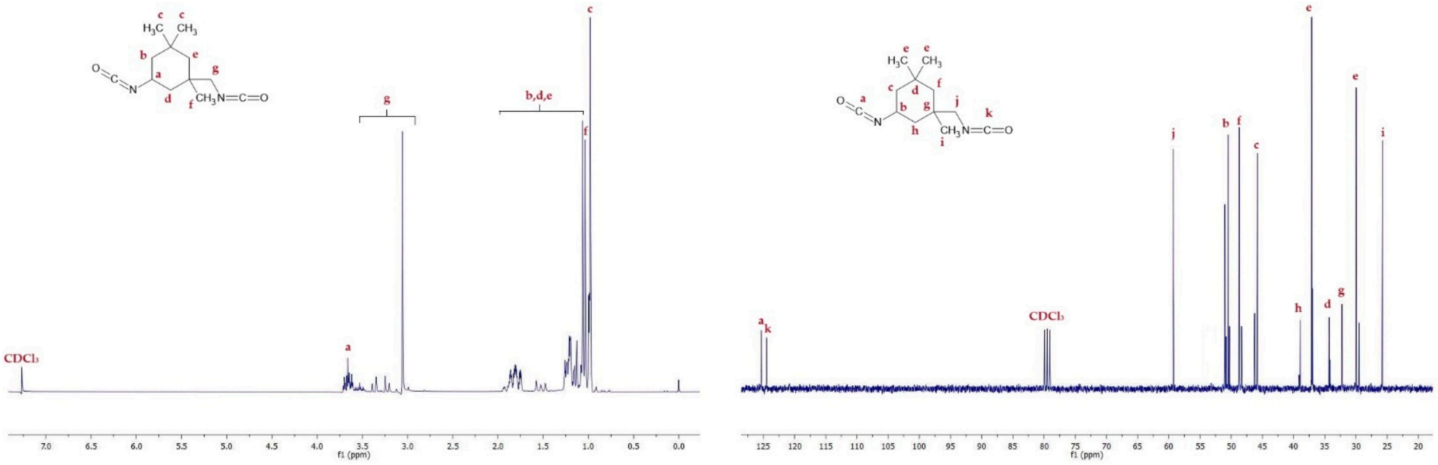

a) b)

Fig.8..

Figure S8. The NMR spectra of isophorone diisocyanate: a) ^{1}H NMR b) ^{13}C NMR.

Table S15. ^{1}H NMR signals of isophorone diisocyanate.

Signal Symbol	Hydrogen Atom	Multiplicity	Number of Protons	Chemical Shift (ppm)
a	-CH<	m	1	3.45-376
b	-CH2-	m	2	1.06-1.30
c	CH3-	m	6	0.98
d	-CH2-	m	2	1.06-1.30
e	-CH2-	m	2	1.06-1.30
f	CH3-	m	3	1.03
g	-CH2-	s and q	2	3.02-3.40

Table S16. ^{13}C NMR signals of isophorone diisocyanate.

Signal Symbol	Carbon Atom	Chemical Shift (ppm)
a	O=C=N-	125
b	-CH<	50
c	-CH2-	46
d	>C<	34
e	CH3-	30 and 37
f	-CH2-	48
g	>C<	32
h	-CH2-	39
i	CH3-	27
j	-CH2-	59
k	O=C=N-	124

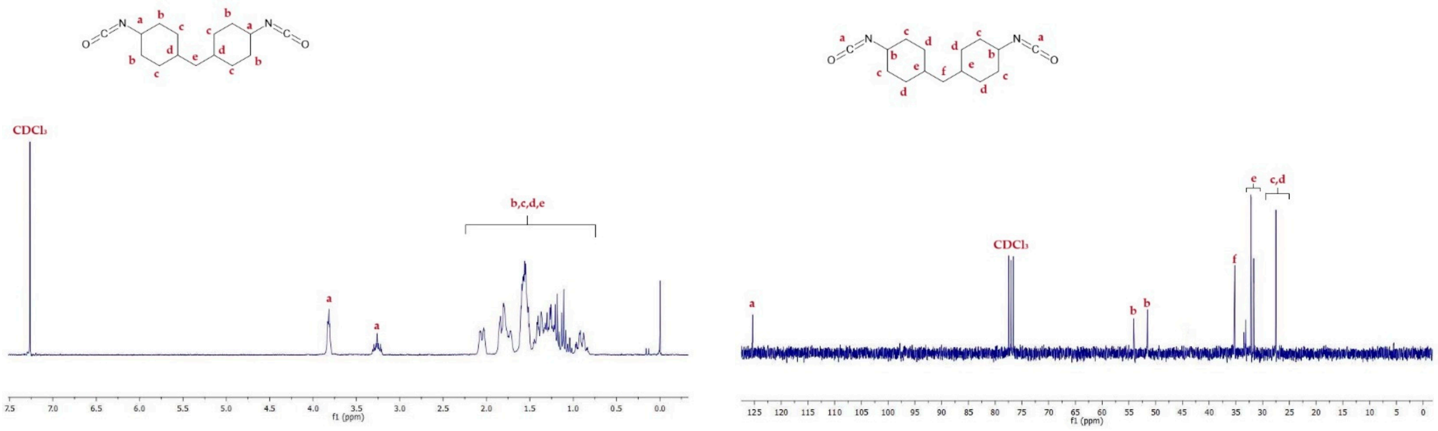

a) b)

Fig.9.

Figure S9. The NMR spectra of dicyclohexylmethane 4,4'-diisocyanate: a) ^{1}H NMR b) ^{13}C NMR.

Table S17. ^{1}H NMR signals of dicyclohexylmethane 4,4'-diisocyanate.

Signal Symbol	Hydrogen Atom	Multiplicity	Number of Protons	Chemical Shift (ppm)
a	-CH<	m	2	3.19-3.35 and 3.81
b	-CH$_2$-	m	8	0.77-2.12
c	-CH$_2$-	m	8	0.77-2.12
d	-CH<	m	2	0.77-2.12
e	-CH$_2$-	m	2	0.77-2.12

Table S18. ^{13}C NMR signals of dicyclohexylmethane 4,4'-diisocyanate .

Signal Symbol	Carbon Atom	Chemical Shift (ppm)
a	O=C=N-	125
b	-CH<	51 and 54
c	-CH$_2$-	28
d	-CH$_2$-	28
e	-CH<	31-34
f	-CH$_2$-	35

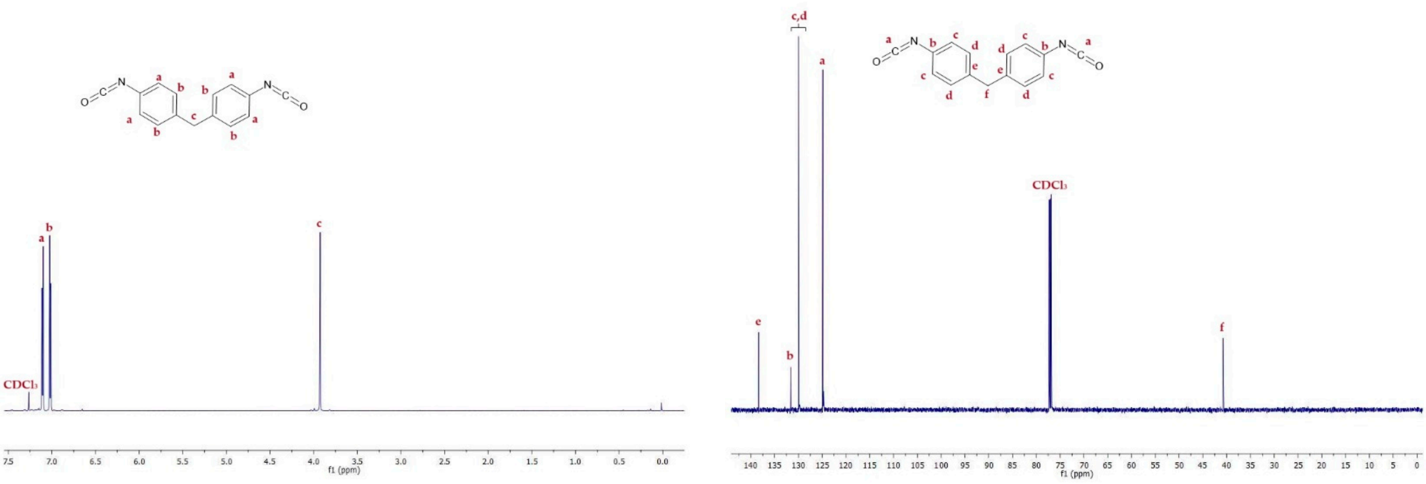

a) b)

Figure S10. The NMR Spectra of 1,1′-methylenebis(4-isocyanatobenzene) : a) ^{1}H NMR b) ^{13}C NMR.

Table S19. ^{1}H NMR signals of 1,1′-methylenebis(4-isocyanatobenzene).

Signal Symbol	Hydrogen Atom	Multiplicity	Number of Protons	Chemical Shift (ppm)
a	-CH-	s	4	3.93
b	-CH-	d	4	6.98-7.05
c	$-CH_2-$	d	2	7.07-7.15

Table S20. ^{13}C NMR signals of 1,1′-methylenebis(4-isocyanatobenzene).

Signal Symbol	Carbon Atom	Chemical Shift (ppm)
a	O=C=N-	125
b	-C<	131
c	-CH-	129
d	-CH-	129
e	-C<	138
f	$-CH_2-$	41

Table S21. ^{1}H NMR signals of QA8+IPDI and QA10+IPDI monomers.

Signal Symbol	Hydrogen Atom	Multiplicity	Number of Protons	Chemical Shift (ppm)
a	$C\underline{H_3}$-C=	s	6	1.94
b	=$C\underline{H_2}$	2m	4	5.67 and 6.12
c	-O-$C\underline{H_2}$-CH_2-N^+-	m	4	4.50-4.75
d	-O-CH_2-$C\underline{H_2}$-N^+-	m	4	3.80-4.30
e	-N^+-$C\underline{H_3}$	s	6	3.30-3.70
f	-O-CH_2-$C\underline{H_2}$-N^+-	m	4	3.80-4.30
g	-O-$C\underline{H_2}$-CH_2-N^+-	m	4	4.50-4.75
h	-N$\underline{H}$-C=O	d and t	2	6.75 and 6.93
i	-N^+-$C\underline{H_2}$-CH_2-$(CH_2)_{5(or\ 7)}$-CH_3 [1]	m	4	3.30-3.70
j	-N^+-CH_2-$C\underline{H_2}$-$(CH_2)_{5(or\ 7)}$-CH_3 [1]	m	4	1.55-1.71
k	-N^+-CH_2-CH_2-$(C\underline{H_2})_{5(or\ 7)}$-$CH_3$ [1]	m	20/28[2]	1.25-1.32
l	-N^+-CH_2-CH_2-$(CH_2)_{5(or\ 7)}$-$C\underline{H_3}$ [1]	m	6	0.86
m	-$C\underline{H_2}$-NH-	m	2	2.89
n	$C\underline{H_3}$-C<	m	3	0.86-1.90
o	$C\underline{H_3}$-C<	m	6	0.86-1.90
p	-$C\underline{H_2}$-	m	2	0.86-1.90
r	-$C\underline{H_2}$-	m	2	0.86-1.90
s	-$C\underline{H_2}$-	m	2	0.86-1.90
t	>C$\underline{H}$-NH-	m	1	3.30-3.70

[1] -N^+-CH_2-CH_2-$(CH_2)_5$-CH_3 corresponds to QA8+IPDI, where -N^+-CH_2-CH_2-$(CH_2)_7$-CH_3 corresponds to QA10+IPDI; [2] 20 corresponds to QA8+IPDI and 28 corresponds to QA10+IPDI

Table S22. ^{1}H NMR signals of QA8+CHMDI and QA10+CHMDI monomers.

Signal Symbol	Hydrogen Atom	Multiplicity	Number of Protons	Chemical Shift (ppm)
a	$\mathrm{C\underline{H}_3\text{-}C=}$	s	6	1.94
b	$\mathrm{=C\underline{H}_2}$	2m	4	5.67 and 6.12
c	$\mathrm{\text{-}O\text{-}C\underline{H}_2\text{-}CH_2\text{-}N^+\text{-}}$	m	4	4.50-4.75
d	$\mathrm{\text{-}O\text{-}CH_2\text{-}C\underline{H}_2\text{-}N^+\text{-}}$	m	4	3.80-4.30
e	$\mathrm{\text{-}N^+\text{-}C\underline{H}_3}$	s	6	3.30-3.70
f	$\mathrm{\text{-}O\text{-}CH_2\text{-}C\underline{H}_2\text{-}N^+\text{-}}$	m	4	3.80-4.30
g	$\mathrm{\text{-}O\text{-}C\underline{H}_2\text{-}CH_2\text{-}N^+\text{-}}$	m	4	4.50-4.75
h	$\mathrm{\text{-}N\underline{H}\text{-}C=O}$	m	2	6.27 and 6.44
i	$\mathrm{\text{-}N^+\text{-}C\underline{H}_2\text{-}CH_2\text{-}(CH_2)_{5(or\ 7)}\text{-}CH_3}$ [1]	m	4	3.30-3.70
j	$\mathrm{\text{-}N^+\text{-}CH_2\text{-}C\underline{H}_2\text{-}(CH_2)_{5(or\ 7)}\text{-}CH_3}$ [1]	m	4	1.55-1.71
k	$\mathrm{\text{-}N^+\text{-}CH_2\text{-}CH_2\text{-}(C\underline{H}_2)_{5(or\ 7)}\text{-}CH_3}$ [1]	m	20/28[2]	1.25-1.32
l	$\mathrm{\text{-}N^+\text{-}CH_2\text{-}CH_2\text{-}(CH_2)_{5(or\ 7)}\text{-}C\underline{H}_3}$ [1]	m	6	0.85
m	$\mathrm{\text{-}C\underline{H}_2\text{-}}$	m	8	0.85-1.94
n	$\mathrm{\text{-}C\underline{H}_2\text{-}}$	m	8	0.85-1.94
o	$\mathrm{\text{-}NH\text{-}C\underline{H}}$<	m	2	3.30-3.70
p	$\mathrm{\text{-}C\underline{H}_2\text{-}}$	m	2	0.85-1.94
r	$\mathrm{\text{-}C\underline{H}\text{-}C}$<	m	2	0.85-1.94

[1] $\mathrm{\text{-}N^+\text{-}CH_2\text{-}CH_2\text{-}(CH_2)_5\text{-}CH_3}$ corresponds to QA8+CHMDI, where $\mathrm{\text{-}N^+\text{-}CH_2\text{-}CH_2\text{-}(CH_2)_7\text{-}CH_3}$ corresponds to QA10+CHMDI; [2] 20 correspods to QA8+CHMDI and 28 correspods to QA10+CHMDI

Table S23. ^{1}H NMR signals of QA8+MDI and QA10+MDI monomers.

Signal Symbol	Hydrogen Atom	Multiplicity	Number of Protons	Chemical Shift (ppm)
a	$C\underline{H_3}$-C=	s	6	1.92
b	=$C\underline{H_2}$	2m	4	5.61 and 6.06
c	-O-$C\underline{H_2}$-CH_2-N^+-	m	4	4.30-4.70
d	-O-CH_2-$C\underline{H_2}$-N^+-	m	4	3.90-4.24
e	-N^+-$C\underline{H_3}$	s	6	3.27
f	-O-CH_2-$C\underline{H_2}$-N^+-	m	4	3.90-4.24
g	-O-$C\underline{H_2}$-CH_2-N^+-	m	4	4.30-4.70
h	-N$\underline{H}$-C=O	m	2	6.76
i	-N^+-$C\underline{H_2}$-CH_2-$(CH_2)_5$-CH_3 [1]	m	4	3.44
j	-N^+-CH_2-$C\underline{H_2}$-$(CH_2)_5$-CH_3 [1]	m	4	1.50-1.80
k	-N^+-CH_2-CH_2-$(C\underline{H_2})_5$-CH_3 [1]	m	20/28 [2]	1.00-1.40
l	-N^+-CH_2-CH_2-$(CH_2)_5$-$C\underline{H_3}$ [1]	m	6	0.85-0.88
m	-C$\underline{H}$- (Ar)	d	4	7.40-7.70
n	-C$\underline{H}$- (Ar)	d	4	7.00-7.20
o	-$C\underline{H_2}$-	m	2	3.77

[1] -N^+-CH_2-CH_2-$(CH_2)_5$-CH_3 corresponds to QA8+MDI, where -N^+-CH_2-CH_2-$(CH_2)_7$-CH_3 corresponds to QA10+MDI; [2] 20 correspods to QA8+MDI and 28 correspods to QA10+MDI

Table S24. ^{13}C NMR signals of QA8+IPDI and QA10+IPDI monomers.

Signal Symbol	Carbon Atom	Chemical Shift (ppm)
a	$\underline{C}H_3$-C=	19
b	$\underline{C}H_2$=	127
c	CH_2=$\underline{C}$<	135
d	-$\underline{C}$OO-	166
e-h	-$\underline{C}H_2$-	58-66
i	-N^+-$\underline{C}H_3$	50
j	-N^+-$\underline{C}H_2$-$(CH_2)_{6(or\ 8)}$-CH_3 [1]	58-66
k	-N^+-CH_2-$(\underline{C}H_2)_{6(or\ 8)}$-$CH_3$ [1]	23-46
l	-N^+-CH_2-$(CH_2)_{6(or\ 8)}$-$\underline{C}H_3$ [1]	14
m	-NH-$\underline{C}$=O	155
n	$>\underline{C}$H-NH-	58-66
o	-CH-$\underline{C}H_2$-C<	58-66
p	$>\underline{C}(CH_3)_2$	23-46
q	$>C(\underline{C}H_3)_2$	23-46
r	$>C$-$\underline{C}H_2$-C<	23-46
s	$>\underline{C}(CH_3)$-	23-46
t	-CH-$\underline{C}H_2$-C<	23-46
u	$>C(\underline{C}H_3)$-	23-46
w	-$\underline{C}H_2$-NH-	58-66

[1] -N^+-CH_2-$(CH_2)_6$-CH_3 corresponds to QA8+IPDI, where -N^+-CH_2-$(CH_2)_8$-CH_3 corresponds to QA10+IPDI

Table S25. ^{13}C NMR signals of QA8+CHMDI and QA10+CHMDI monomers

Signal Symbol	Carbon Atom	Chemical Shift (ppm)
a	$\underline{C}H_3$-C=	19
b	$\underline{C}H_2$=	128
c	CH_2=$\underline{C}$<	136
d	-$\underline{C}$OO-	166
e-h	-$\underline{C}H_2$-	55-66
i	N^+-$\underline{C}H_3$	50
j	-N^+-$\underline{C}H_2$-$(CH_2)_{6(or\ 8)}$-CH_3 [1]	55-66
k	-N^+-CH_2-$(\underline{C}H_2)_{6(or\ 8)}$-$CH_3$ [1]	23-35
l	-N^+-CH_2-$(CH_2)_{6(or\ 8)}$-$\underline{C}H_3$ [1]	14
m	-NH-$\underline{C}$=O	155
n	>$\underline{C}$H-NH-	51
o	-$\underline{C}H_2$-	23-35
p	-$\underline{C}H_2$-	23-35
q	>$\underline{C}$H-	23-35
r	-$\underline{C}H_2$-	49

[1] -N^+-CH_2-$(CH_2)_6$-CH_3 corresponds to QA8+CHMDI, where -N^+-CH_2-$(CH_2)_8$-CH_3 corresponds to QA10+CHMDI

Table S26. ^{13}C NMR signals of QA8+MDI and QA10+MDI monomers.

Signal Symbol	Carbon Atom	Chemical Shift (ppm)
a	$\underline{C}H_3$-C=	19
b	$\underline{C}H_2$=	126
c	$CH_2=\underline{C}<$	138
d	-$\underline{C}$OO-	166
e-h	-$\underline{C}H_2$-	53-64
i	N^+-$\underline{C}H_3$	49
j	-N^+-$\underline{C}H_2$-$(CH_2)_{6(or\ 8)}$-CH_3 [1]	53-64
k	-N^+-CH_2-$(\underline{C}H_2)_{6(or\ 8)}$-$CH_3$ [1]	23-32
l	-N^+-CH_2-$(CH_2)_{6(or\ 8)}$-$\underline{C}H_3$ [1]	14
m	-NH-$\underline{C}$=O	153
n	>$\underline{C}$-NH-	138
o	-$\underline{C}$H-	120
p	-$\underline{C}$H-	128
q	>$\underline{C}$-	136
r	-$\underline{C}H_2$-	42

[1] -N^+-CH_2-$(CH_2)_6$-CH_3 corresponds to QA8+MDI, where -N^+-CH_2-$(CH_2)_8$-CH_3 corresponds to QA10+MDI

Table S27. The interpretation of FTIR spectra of QA8+IPDI and QA10+IPDI monomers.

Chemical bond	Intensity [1]	Wavenumber (cm^{-1})
N-H	w	3215
CH_3	w	3064
$=CH_2$	m	2940
CH_2, CH_3	m	2922 and 2855
C=O	s	1714
C=C	w	1638
NH (urethane)	m	1534
CH_2, CH_3	m	1455
C-N	m	1238 and 1160
C-O-C	m	1044 and 1008
$C-N^+$	m	941

[1] The intensity of the absorption bands in the FTIR spectra was referred to as strong (s), medium (m), and weak (w).

Table S28. The interpretation of FTIR spectra of QA8+CHMDI and QA10+CHMDI monomers.

Chemical bond	Intensity [1]	Wavenumber (cm^{-1})
N-H	w	3215
CH_3	w	3046
$=CH_2$	m	2944
CH_2, CH_3	m	2932 and 2848
C=O	s	1718
C=C	w	1628
NH (urethane)	m	1536
CH_2, CH_3	m	1460
C-N	m	1228 and 1152
C-O-C	m	1044 and 1008
$C-N^+$	m	941

[1] The intensity of the absorption bands in the FTIR spectra was referred to as strong (s), medium (m), and weak (w).

Table S29. The interpretation of FTIR spectra of QA8+MDI and QA10+MDI monomers.

Chemical bond	Intensity [1]	Wavenumber (cm^{-1})
N-H	w	3215
CH_3	w	3066
$=CH_2$	m	2936
CH_2, CH_3	m	2929 and 2850
N=C=O [2]	s	2250
C=O	s	1716
C=C	w	1638
C=C (Ar)	w	1613
NH (urethane)	m	1529
CH_2, CH_3	m	1455
C-N	m	1238 and 1160
C-O-C	m	1077 and 1010
$C\text{-}N^+$	m	943

[1] The intensity of the absorption bands in the FTIR spectra was referred to as strong (s), medium (m), and weak (w).
